# Watershed memory amplified the Oroville rain-on-snow flood of February 2017

**DOI:** 10.1093/pnasnexus/pgac295

**Published:** 2022-12-16

**Authors:** Kayden Haleakala, W Tyler Brandt, Benjamin J Hatchett, Dongyue Li, Dennis P Lettenmaier, Mekonnen Gebremichael

**Affiliations:** Department of Civil and Environmental Engineering, University of California Los Angeles, Los Angeles, CA 90095, USA; Center for Western Weather and Water Extremes, Scripps Institution of Oceanography, La Jolla, CA 92093, USA; Center for Western Weather and Water Extremes, Scripps Institution of Oceanography, La Jolla, CA 92093, USA; Division of Atmospheric Sciences, Desert Research Institute, Reno, NV 89512, USA; Department of Geography, University of California Los Angeles, Los Angeles, CA 90095, USA; Department of Geography, University of California Los Angeles, Los Angeles, CA 90095, USA; Department of Civil and Environmental Engineering, University of California Los Angeles, Los Angeles, CA 90095, USA

**Keywords:** rain-on-snow, flood, Oroville Dam, terrestrial water input, antecedent conditions

## Abstract

Mountain snowpacks are transitioning to experience less snowfall and more rainfall as the climate warms, creating more persistent low- to no-snow conditions. This precipitation shift also invites more high-impact rain-on-snow (ROS) events, which have historically yielded many of the largest and most damaging floods in the western United States. One such sequence of events preceded the evacuation of 188,000 residents below the already-damaged Oroville Dam spillway in February 2017 in California’s Sierra Nevada. Prior studies have suggested that snowmelt during ROS dramatically amplified reservoir inflows. However, we present evidence that snowmelt may have played a smaller role than previously documented (augmenting terrestrial water inputs by 21%). A series of hydrologic model experiments and subdaily snow, soil, streamflow, and hydrometeorological measurements demonstrate that direct, “passive” routing of rainfall through snow, and increasingly efficient runoff driven by gradually wetter soils can alternatively explain the extreme runoff totals. Our analysis reveals a crucial link between frequent winter storms and a basin’s hydrologic response—emphasizing the role of soil moisture “memory” of within-season storms in priming impactful flood responses. Given the breadth in plausible ROS flood mechanisms, this case study underscores a need for more detailed measurements of soil moisture along with in-storm changes to snowpack structure, extent, energy balance, and precipitation phase to address ROS knowledge gaps associated with current observational limits. Sharpening our conceptual understanding of basin-scale ROS better equips water managers moving forward to appropriately classify threat levels, which are projected to increase throughout the mid-21st century.

Significance Statement Extreme rain-on-snow can cause severe flooding. However, current observational networks can mislead efforts to understand the key elements of such events (rainfall, snowmelt, and how water travels through snow). We demonstrate this ambiguity in a case study of an impactful rain-on-snow event in California’s northern Sierra Nevada during 2017. Results suggest snowmelt played a smaller role in augmenting runoff than previously documented. Rather, consecutive storms gradually saturated soils to amplify the runoff response to rainfall and snowmelt. Our alternative explanation calls for improving measurement and modeling capacities, as better forecasting should follow better “water accounting” of these impactful events. Such improvements are critical as the climate shifts toward increasingly dangerous rain-on-snow events over more transient snow-covered areas.

## Introduction

Mountain rain-on-snow (ROS) produces some of the largest and most damaging floods in the western United States (1–3). In California’s Sierra Nevada, ROS flooding commonly occurs due to landfalling atmospheric rivers (ARs). ARs bring warm, humid, and windy conditions with prolonged precipitation that possess anomalously high snow levels over vast, typically snowfall-dominated landscapes (4–8). Storm sequencing can amplify or dampen the risk of ROS—with ephemeral snowpacks the most at-risk to rapid melt. Climate warming elevates the flood risk by increasing precipitation extremes (9, 10) and shifting precipitation phase from snow to rain over snowpack—which is in a continued upslope retreat (11). These changes make ROS a transient, but immediate flood hazard that requires skillful forecasts to minimize. Accurate modeling and forecasting of ROS in turn depends on a robust, physically based conceptualization of flood generation, both on a storm-by-storm basis and in the broader context of how the wet season unfolds. Improving societal ROS flood preparedness therefore requires two connected components of predictive understanding.

First, the degree to which snowmelt amplifies runoff during ROS is crucial yet highly variable (12). Snowmelt contributions are often quantified by comparing the snowmelt volume to the sum of rainfall and snowmelt, or terrestrial water input (TWI), which can range from 0 (13, 14) to 60% (15). While rainfall primarily drives TWI (12), even small snowmelt contributions (e.g., ∼10%) at unfavorable times or locations can dictate whether or not a water engineering emergency occurs. Snowmelt is the product of the energy balance, and can only begin once energy inputs exceed the snowpack’s heat capacity (i.e., its cold content) (16). Once cold content is satisfied, meteorological conditions can drive a positive energy balance through high humidity (i.e., latent heat and longwave radiation), air temperature (i.e., sensible heat), and wind speed (which enhances turbulent mixing) that induces snowmelt (16). Case studies indicate that turbulent and longwave radiative fluxes during extreme ROS can dominate snowmelt. Several examples of these “active” (12) contributions to TWI range between 21 to 56% in the United States Pacific Northwest (17), 13 to 26% in the Swiss Alps (18), 25% in the California Sierra Nevada (19), and 2 to 60% in Black Forest, Germany (15, 20). These large ranges indicate the diversities in both storm meteorological drivers and snowpack states, which vary considerably across elevation, aspect, and vegetation (4, 13, 15, 17, 18, 21–24). Other energy balance components can also be important, including the ground heat flux (25) and the heat advection from rainfall (26)—two sometimes neglected terms (16). Large-sample studies of ROS events (as opposed to case studies) show that net radiation usually dominates snowmelt (1, 27), which only tends to contribute less than 30% to TWI (23, 28, 29). Snowpacks in such ROS events are considered “active,” meaning that melt contributions to TWI exceed 10%. Snowmelt from “passive” snowpacks contribute less than 10% to TWI—a nominal threshold representing the small yet inevitable heat advection delivered to snow during rainfall (12, 22). In the Sierra Nevada, rainfall contributions dominate TWI (∼77 to 95%) (4, 30).

The second component to predictive understanding of ROS involves how liquid travels through snow, which affects runoff timing and volume. Two flow regimes broadly characterize this. First, rain and/or snowmelt may flow as a uniform wetting front (“matrix flow”), propagating vertically and uniformly through the snowpack—a relatively slow, steady process. While matrix flow is commonly observed in shallow, mature, or melting snow (31–33), it is far from ubiquitous (30, 31, 34, 35), especially in the maritime midwinter snowpacks of the Sierra Nevada. Nonetheless, most physically-based models to date simulate matrix flow (or saturation excess within snow layers) to estimate TWI (36–39). The second flow regime is preferential flow, which consists of pathways that collect and route liquid through the snowpack (40–42). Rainfall (22) or a warm (i.e., low cold content) snowpack (42, 43) can develop high-conductivity flow-paths by “connecting the plumbing.” Preferential flow enables a “passive” response during ROS, quickly routing rainfall vertically or laterally through snow into streams, bypassing much of the snow matrix (13, 33, 44), and advancing TWI timing from weeks to days (42)—sometimes as fast as 6 to 7 m hour^−1^ (22, 45). Quickly bypassing the snow matrix theoretically limits some of the sensible heat exchange from rain to below-0°C snow (12, 16). On the other hand, crusts within a snowpack can suspend liquid and delay snowpack outflow by hours, allowing more sensible heat (24, 40, 46). Importantly, the spatial variability within snowpack layering (both vertically and horizontally) drives how each flow regime modulates runoff timing and volume (12, 13, 22, 23).

Given these nuances, physically-grounded conceptualizations of ROS are crucial to an accurate and precise forecast. However, this is challenged by a lack of observations capable of detailing the above-mentioned mechanisms in space and time, and at scale. Matrix and preferential flow regimes co-exist and evolve (31, 32, 35). Few mountain locations monitor energy exchanges directly (47, 48) for accurate snowmelt estimates. This leaves important energy balance drivers parameterized or unverifiable. Standard observations from long-standing networks (49, 50) (e.g. daily telemetered precipitation, temperature, and snow depth and/or water equivalent) may provide biased precipitation, mischaracterize precipitation phase (51), and only provide a bulk snowpack representation. In turn, the use and validation of hydrologic models necessary for ROS flood forecasting in snow-dominated basins have important processes unresolved or misrepresented through calibration. As a result, our predictive understanding is vulnerable to adopting inappropriate hydrologic concepts.

**Fig. 1.**
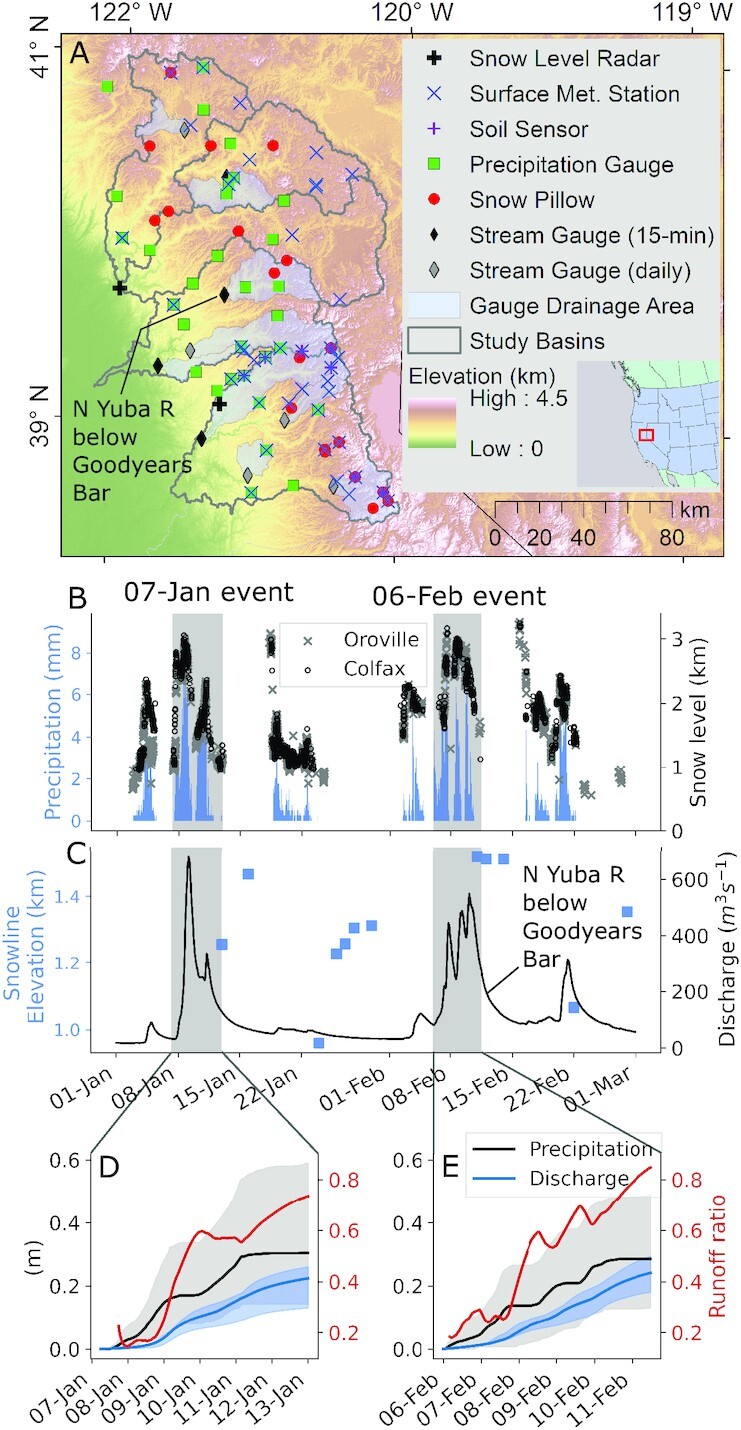
(A) Snow, river, and hydrometeorological monitoring stations in the Feather (North Fork, east branch of North Fork, and Middle Fork), Yuba, and American River basins. Gray-shaded areas drain to each US Geological Survey (USGS) stream gauge, which report daily or 15-minute measurements. (B) Median hourly incremental precipitation from the network in (A), and 10-minute brightband height (BBH) from snow level radars in January through February 2017. The 7 January and 6 February storm sequences are gray shaded. (C) Daily regional snowline elevation [calculated using Moderate Resolution Imaging Spectroradiometer (MODIS) fractional snow-covered area], and 15-minute stream discharge at USGS gauge 11413000. Cumulative discharge and precipitation median and range are shown for the (D) 7J and (E) 6F storm events, with median runoff ratios shown in red.

This study reveals an ambiguity in how we observe, simulate, and interpret the role of snowpacks in ROS flooding. We investigate the storms involving ROS that coincided with the 2017 Oroville Dam spillway failure in California’s northern Sierra Nevada that prompted an evacuation of 188,000 people. While snowmelt is understood to have contributed strongly to TWI in this event, we present evidence that argues for an alternative explanation for the extreme runoff—namely, antecedent soil moisture. Using subdaily snow, soil, streamflow, and hydrometeorological measurements in the Feather, Yuba, and American River basins (hereafter “study basins,” Fig. 1A), with supporting satellite and atmospheric reanalyses and hydrologic modeling experiments, we show how preferential flow through snow along with progressively saturated soils from previous storm cycles provide a viable alternative explanation. This “integrated” description of the same event carries diverging implications regarding where future efforts to improve forecast skill of extreme events can be most effectively targeted. Such innovations should constrain the key mechanisms driving changes in landscape saturation and snowpack liquid water content. Facing a climate with more frequent and severe ARs impinging on snowpacks susceptible to active ROS, converging on an unambiguous concept of mountain flood generation is crucial for future flood preparedness.

## Rain-on-Snow Events during Winter 2016/2017

Beginning in 2011, the Sierra Nevada experienced one of the most severe (52) droughts in recorded history prior to water year (WY) 2017—a record precipitation year that broke the meteorological drought. The northern Sierra Nevada accumulated over 2,200 mm of precipitation from 49 landfalling ARs between 1 October 2016 and 12 April 2017 (53). A water resource tradeoff ensued: some major reservoirs filled, quelling the hydrological drought (54), while others flooded (55, 56). In January and February, eight AR families made landfall in northern California (57), bringing several distinct, prolonged spells of precipitation (55, 56) (Fig. 1B). Two storm sequences—one from 7 to 12 January (hereafter 7J) and the other from 6 to 12 February (58) (6F)—were accompanied by high snow levels (Fig. 1B) and prominent peaks in unimpaired river discharge (Fig. 1C).

**Fig. 2.**
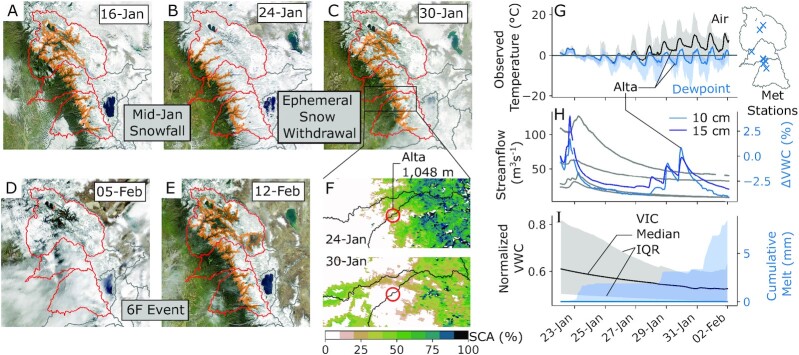
True-color evolution of the snow cover spans snowfall (A and B) and its ablation in late January (C) preceding the February 2017 ROS event (D and E). Orange contours show the regional snowline elevation as in Fig. 1C. The Alta station monitors both weather and soil, and is located in this ephemeral snow region transitioning from snow-covered to snow-free (F). Air and dewpoint temperatures at surface stations in this ephemeral elevation range (960 to 1,312m) gradually approach and exceed 0°C (G), with corresponding fluctuations in Alta’s soil moisture despite no responses in subdaily streamflow (H). These indicate that the ephemeral snow melted (as opposed to sublimated), a result corroborated by distributed model estimates of minimal snowmelt and soil moisture response (I).

The 7J sequence accumulated 329 mm of precipitation (median across precipitation gauges in the study basins), and 224 mm of coincident discharge (median across stream gauges; Fig.   1D). Beginning on 7 January 0730Z, snow levels rose rapidly, peaking at 3,059 m (above the highest point in all three basins) on 8 January 1350Z before falling. A second warm pulse of precipitation brought snow levels back up to 2,364 m (above 98% of the study area) on 11 January 0420Z before declining again. Both snow level rises accompanied peaks in streamflow. The 7J sequence was followed by a relatively cold and modest storm from 13 to 23 January, with 103 mm of median precipitation and lower snow levels averaging 1,319 m (above 35% of the basin). This storm minimally impacted streamflow, but lowered the regional snowline to 960 m (Figs.   1C and 2B).

The 6F event began with a rapid rise in snow levels from 1,915 to 3,169 m between 7 February 0200Z–1400Z (Fig. 1B), lowering gradually. Three consecutive waves of precipitation occurred, each with snow levels above 1,500 m and distinctive streamflow signatures (Fig. 1B and C). The 6F event accumulated 322 mm of median precipitation and 241 mm of median discharge. Streamflow responses, taken together with snow levels above the regional snowline, provide evidence of ROS in both the 7J and 6F events.

Both ROS events had similar synoptic characteristics (Fig. S1 and Table S1). We assessed these during periods when snow levels exceeded 1,600 m. Both 7J and 6F total integrated vapor and heat transport, and average moist static energy (MSE) gradients between 500- and 850-hPa, were within ±4% (Table S1). This suggests the atmospheric conditions during both events sustained similar degrees of heat and moisture advection and static stability (59). Nonetheless, surface stations indicate that the 6F event was generally warmer, particularly below 1,200 m, while the 7J event was more wind-driven (Table S1; corroborated by reanalysis-derived wind fields; Fig. S1). One notable difference between the events was a cold front during 7J, which lowered snow levels by ∼1,250 m (Fig. S1 and 1B). Given a greater runoff-to-precipitation ratio (0.85) and a slower decline in snow levels in the 6F event (Fig. 1B, D, and E), a reasonable hypothesis may be that snowmelt augmented the 6F hydrograph, which preceded the spillway incident at Lake Oroville (19, 55).

## Snow as a Passive Conduit for Rainfall

A previous case study inferred the importance of snowmelt in the 6F event by noting that while precipitation only ranked the 9th-highest (on record) in the Feather River basin, runoff, in contrast, was ranked 2nd-highest (19). The authors reasoned the extreme discharge was only possible with supplemental snowmelt. They estimated that snowmelt augmented TWI by ∼37% relative to rainfall alone, which was supported by observations of daily snow water equivalent (SWE) decreases at snow pillows, an upslope migrating snow cover, and subsequent declines in spatially distributed SWE between 24 January and 12 February (19). While high runoff ratios (Fig.   1D and E) and above-0°C temperatures (Table S1) indeed suggest snowmelt amplified TWI, three lines of evidence suggest a different plausible interpretation of events.

First, while we do not contest that snowlines retreated during the study period, we do contest how much of the retreat can be attributed to ROS versus ablation unrelated to the 6F storm event. SWE estimates (blending station interpolation, modeled reconstruction, and satellite-derived fractional snow-covered area, or fSCA) on 24 January and 12 February were used by the previous study to calculate ROS snowmelt (19). We note that the image date selection—which bracketed the storm—was reasonable, given restrictions from both cloud cover and/or large zenith angles (60). However, a visual inspection of all available images reveals a substantial snowline withdrawal before the 6F event occurred—from 960 m on 24 January to 1,312 m on 30 January (Fig. 2B and C). Neither streamflow (Fig. 2H) nor snow pillow SWE (Fig. 4C) changed during this time. However, observed soil moisture increased in the elevation range where snow disappeared (Fig. 2F to H). This, along with the estimates of snowmelt and soil moisture responses from the Variable Infiltration Capacity [VIC (61)] hydrologic model (Fig. 2I) suggest that the ephemeral snow withdrawal may have contributed (62) to the 6F antecedent soil conditions. The extensive fSCA on 24 January therefore may have led to a misinterpretation by the previous study that the subsequent 6F event produced a larger snowmelt contribution to ROS flooding than actually occurred. The size of this bias depends on the difference between watershed volumes of SWE on 24 January and 5 February.

**Fig. 3.**
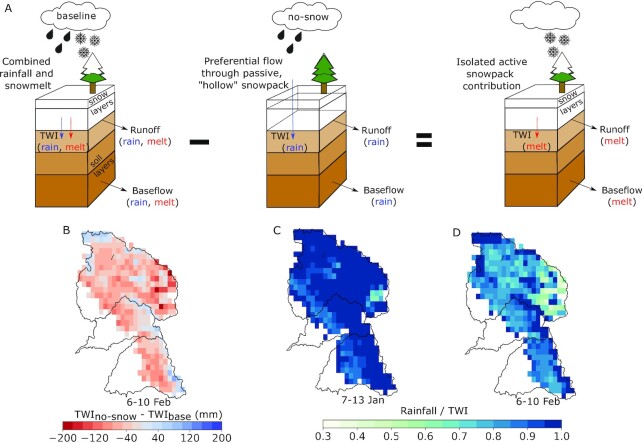
(A) Conceptual diagram of a model experiment to isolate the role of “active” snowpack during the 6F ROS event. Simulating “no-snow” with the same baseline liquid precipitation effectively illustrates preferential flow through a “passive” snowpack. The difference in accumulated TWI between these two scenarios (B) represents the role of snowpack in augmenting TWI. Baseline rainfall to TWI ratios in the (C) 7J and (d) 6F events show a dominant rainfall contribution to TWI.

Second, using a distributed energy balance and a simplified implementation of preferential flow, we estimate that snowmelt contributions to TWI were lower than previously calculated [relative to 76 mm, or ∼25% of TWI in the Feather River basin (19)]. To approximate the role of snowmelt in driving TWI and to conceptualize preferential flow in a completely “passive” ROS response to the 6F event, we conducted a “no-snow” experiment that considers only the 6F liquid precipitation. This underestimates TWI by the approximate snowmelt amount and provides a benchmark for assessing the role of active snowpack in the event. We estimate snowmelt counterfactually by taking the total TWI difference between preferential flow and baseline simulations of the event (Fig. 3A and B). In the Feather River basin, this difference in TWI (and thus approximate snowmelt volume) is 47 mm and is insensitive to the model’s precipitation partitioning temperature (Fig. S2). This suggests a 38% less “active” snowpack than previously reported [augmenting TWI by 21% compared to 37% (19)]. Importantly, rainfall comprises most of the TWI from both the 7J and 6F events, where 99 and 79% of the snow-covered areas have rainfall contributions to TWI exceeding 75%, respectively (Fig.   3C and D). The regions in which the scenario difference in event-accumulated TWI is non-negative (Fig. 3B) may be interpreted as “passive” or allowing preferential flow, as they indicate no additional snowpack contributions to TWI.

**Fig. 4.**
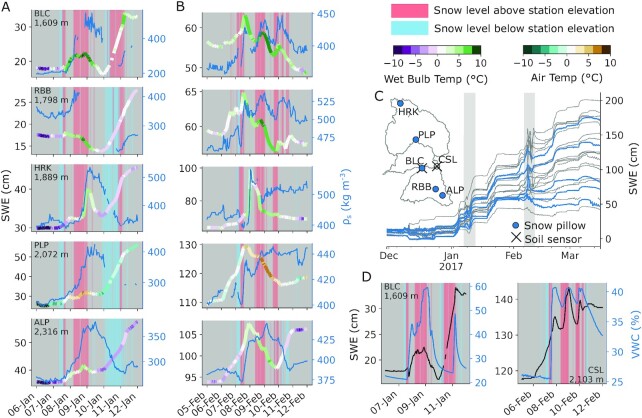
Snow pillow responses to the (A) 7J and (b) 6F ROS events show hourly SWE traces colored by air temperature (or wet-bulb temperature, if humidity measurements are available), and bulk snow density (if snow depth measurements are available). Vertical shading indicates periods when atmospheric snow levels were above or below snow pillow elevations. (C) Snow pillows and storms in (A) and (B) are plotted with respect to the winter 2017 at all snow pillows used in this study. (D) Two locations within our study basins have collocated SWE and soil moisture (≤10 cm depth) during these storm events, showing in-phase responses to rainfall.

**Fig. 5.**
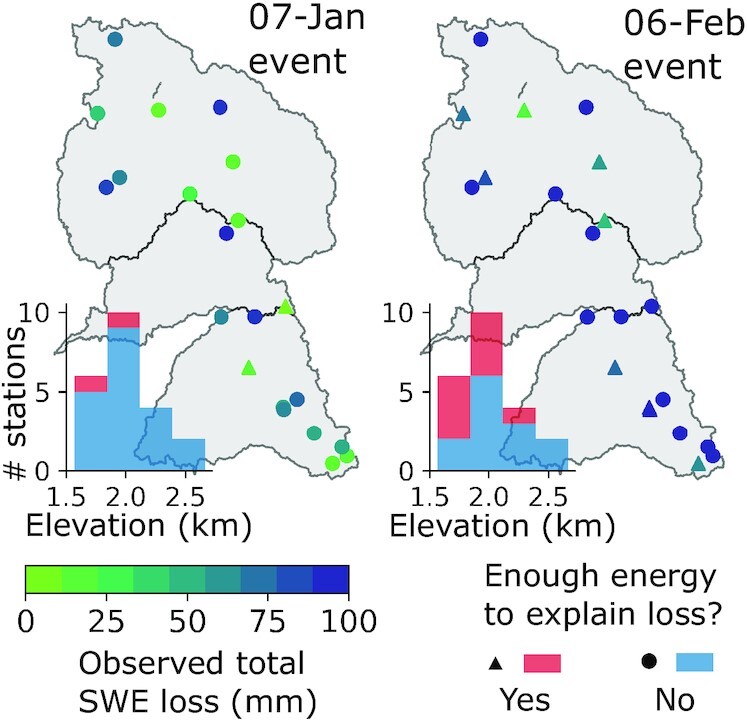
Energy balance approximations at the nearest VIC grid cell to snow pillows indicate whether observed losses in SWE (Fig. 4A and B, and S3 to S5) could be completely explained as snowmelt in the (left) 7J and (right) 6F events. Inset histograms show the elevation distribution of snow pillows.

Finally, a third line of evidence regards “standard” daily measurements (including temperature, snow depth, SWE, and precipitation), which have been used previously to identify and interpret ROS events (11, 29, 63, 64). While this avoids the instrument error- and noise-related problems with subdaily measurements (65), we note that daily timesteps may mask or misrepresent the mechanism(s) generating runoff during ROS. For instance, the 6F event showed widespread declines in daily SWE from 7 to 10 February [Fig. 3 in ref. (19)], which could indicate widespread snowmelt. However, these daily values only correspond to roughly 1200Z, as “daily” values represent a single measurement between 0300 and 0400 local time rather than a 24-hour aggregation of measurements [https://www.cnrfc.noaa.gov/awipsProducts/RNOFSTSWE.php; California Department of Water Resources (DWR), personal communication]. In contrast, hourly SWE data paint a distinctly different picture, exhibiting SWE “pulses” (66) (Figs. 4B and S3 to S5B). Importantly, the SWE rises and falls steeply—in some cases, returning close to the same SWE as when the pulse began. These “pulses” also commence during times of heavy precipitation (6 February 0200Z) and snow density increases (a classic ROS indicator; Figs. 4B and S3 to S5B), and occur when snow levels were above snow pillow elevations, while air/wet-bulb temperatures were above 0ºC. These observations converge on the likely presence of ROS. Given the steep SWE oscillations, rather than assuming this might be accumulating and ablating ice, it could be hypothesized that this is in fact a mass shift due to the exchange of liquid water (i.e., rainfall saturating and draining from the snowpack). Pulses also occur across the snow pillow network (66) earlier in the WY. A similar SWE pulse occurred during the 7J event (Figs.   4A and S3 to S5A), and similar pulses have been observed across the Sierra Nevada during warm storms (67, 68) and during past ROS events (17). Moreover, some of these SWE oscillations occur in-phase with shallow (10 cm or less) collocated soil moisture measurements (Fig. 4D), supporting the concept that “pulses” resemble transient rainfall storage and passage through snow. Importantly, the falling limb of these pulses may not necessary be entirely snowmelt. Our energy balance modeling corroborates this idea with not enough energy available to melt the observed SWE declines at half of the snow pillows (Fig. 5). In summary, our findings support the notion that daily snow pillow observations of ROS may be misleading, and may result in a larger perceived melt contribution to TWI.

We note, however, that this interpretation does not demonstrate that the ROS responses at snow pillows were completely passive. It is possible, for instance, for out-of-phase soil moisture-SWE behaviors during ROS to occur as a result of lower-conductivity soils (Fig. S6). This may be indistinguishable from the SWE-soil moisture trace during snowmelt (69), making it inappropriate to infer active/passive responses from these measurements alone. A lack of observations (e.g., stable isotopes to separate rainfall from snowmelt) leaves this “passive” interpretation open and testable, but out of this study’s scope.

It is also possible that rainfall underestimation makes up for inflated snowmelt contributions to TWI. Quantifying mountain precipitation is a pervasive hydrometeorological challenge (70) tied directly to estimating relative snowmelt contributions to TWI. Applying a simple wind-correction (71) from hourly reanalyses to gridded precipitation raises 7J and 6F precipitation by 6 to 12% (Fig. S7). However, this assumes precise precipitation—which varies spatially compared to the forest-protected clearings that contain precipitation gauges—and accurate wind fields, which tend to be muted in mountains (72). This, and unaccounted orographic enhancement of precipitation (55), appears to make this correction a lower bound (thereby lowering the melt contribution to TWI). However, ground and satellite observations are partial to exposed, flat terrain. Vegetation tends to collect less snowpack in-stand compared to exposed areas (73, 74), yet it can shelter snow from wind-driven turbulent heat exchange, potentially lowering TWI during ROS (12, 17, 75). Beneath-canopy SWE and its in-storm changes are invisible to both satellites and snow pillows. Snow-covered areas in our study basins are dominated by forest (65 to 88%) compared to the meadow settings in which ground observations are collected (11 to 30%, Fig. S8). This may affect (1) the location of the regional snowline (as calculated using fSCA here) and (2) the TWI during ROS inferred from snow pillows (76). Relatively broader forest cover therefore suggests actual snowpack losses across watersheds may be lower (reducing the snowpack contribution to TWI) (74, 77) than what may be implied by in-situ SWE losses. This is supported by our modeling results that account for canopy–snow interactions, indicating lower snowmelt contributions to TWI in the 6F ROS event.

## Soils Connect and Amplify Consecutive Storms

**Fig. 6.**
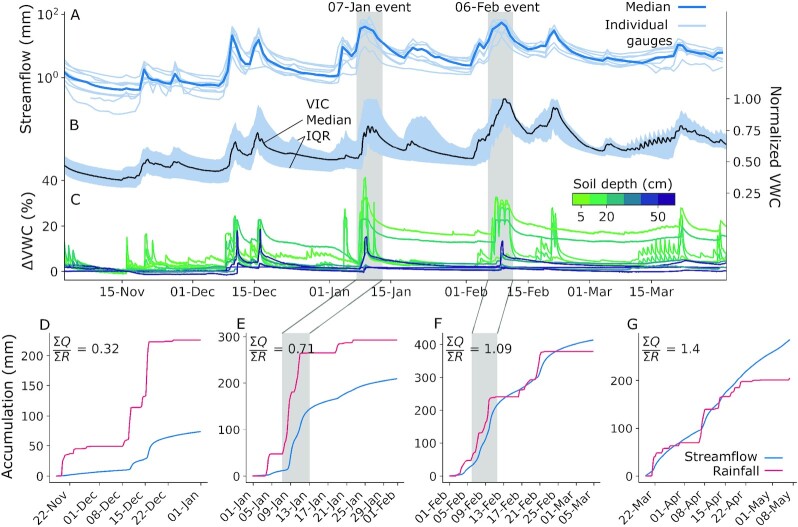
Winter (A) observed daily streamflow at nine gauges and (B) modeled soil moisture in our study basins. (C) Departure in observed soil moisture at high-elevation stations (*n*  = 6 above 1,600 m) from values preceding the 16 November 2016 storm. Note that the deep soil moisture at certain stations (e.g., at CSL) became saturated before this period and, therefore, display no change. (D) Streamflow and estimated rainfall (the liquid portion of total precipitation) accumulations at the stream gauge in the central Feather (USGS gauge 11402000). Rainfall values are aggregated from gridded precipitation over the catchment area upstream of the gauge (Fig.   1A).

What other processes may explain the large ROS runoff if it was not driven by snowmelt? During WY 2017, successive winter storms caused streamflow across the study basins to recess less with the onset of each storm event (Fig. 6A). This indicates an increasingly saturated landscape up until the 6F storm cycle, when log-transformed streamflow levels off. Soil moisture, even in snow-covered areas, echoed the streamflow trajectory. Soils returned to greater moisture levels after each TWI instance as winter progressed (Fig. 6B and C), reflecting greater tendencies to generate runoff from TWI. The increases in “rest” levels of both soil moisture and streamflow suggest that these inputs increase runoff efficiency and baseflow (78). These antecedent conditions are corroborated by steady increases in groundwater levels, as observed from wells in the Yuba and Feather River basins [cf. Figs. 22 and 23 in ref. (79)]. Therefore, we might conclude that the degree of discharge achieved during the 6F storm sequence was facilitated by the 7J sequence and, in turn, due to earlier runoff-generating storm events starting as early as mid-October (56).

Runoff efficiencies increased as WY 2017 progressed, but through which components (rainfall, snowmelt, and/or soil saturation)? We designed two thought experiments to answer this. First, we compared observed accumulated discharge and rainfall, and theorized that large snowmelt contributions should cause runoff to exceed rainfall. Early-season (November–December 2016) rainfall registered relatively little discharge at the central Feather stream gauge (Fig. 6D). Differences between cumulative discharge and rainfall narrowed after the 7J event (Fig.   6E) and narrowed further still after the 6F event (Fig. 6F). But, by the end of February, runoff exceeded rainfall by 9%—an indication that snowmelt may have driven some of the runoff increases. This exceedance is consistent with observed low-elevation SWE losses for which sufficient energy was available for snowmelt (Fig.   5). We also examined a “null case” during the spring to test our thought experiment. As expected, spring discharge amounts strongly exceeded rainfall totals (Fig. 6G) in association with widespread seasonal snowmelt that occurred by late March (Fig. S9). Importantly, the near-linear spring discharge accumulation is distinct from the relatively abrupt accumulations following winter rainfall (Fig. 6D to F). Stream gauges in the Yuba and American River basins exhibited similar results to those in the Feather (Fig. S10), although South Yuba runoff decreased in the spring as a result of minimal snow cover (Fig. S11). We note that melt contributions may not be the only factor in augmenting streamflow; groundwater infiltration or exfiltration can conceivably dampen or amplify streamflow, respectively (78). Additionally, we partition rainfall here using upwind snow level radars in the Sierra Nevada foothills (Fig. 1A). While dynamical and thermodynamical processes cause snow levels to bend downwards with increasing elevation along the windward slopes (80) [biasing the local liquid precipitation fraction (65)], systematically lowering snow levels (Fig. S12) minimally affected our results and interpretation.

**Fig. 7.**
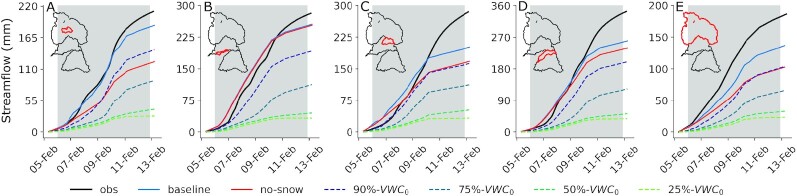
Observed and modeled cumulative streamflow during the 6F ROS event at stream gauges in the (A) Central Feather, (B) South Yuba (USGS gauge 11418500), (C) North Yuba, (D) North Fork American (USGS gauge 11427000), and (E) the Feather River basin (observed full-natural flow into Lake Oroville). Scenarios compare the baseline run to experiments removing the 5 February snowpack (“no-snow”) and those systematically lowering the 5-February soil moisture (“N%-VWC_0_”). Drainage areas are outlined in red.

For our second thought experiment, we considered the effects of drier soils on modeled 6F event runoff. As expected (Fig. 3B), the no-snow scenario produced less runoff from the snow-covered catchments (Fig. 7A, C, and D), indicating non-negligible snowpack contributions that are greatest, for example, at the central Feather River stream gauge (Fig. 7A). However, the baseline snowpack response over drier soils had a much greater runoff impact than the no-snow scenario. At the central Feather River stream gauge, a 10% reduction in the 5 February soil moisture state produced a runoff response within 24% of the no-snow response (Fig. 7A), while drying soils by 25% or more reduced runoff beyond what could be made up by the 6F snowmelt volume. All gauges reflect this response (Fig. 7). The dramatic difference between scenarios confirms the well-known idea that antecedent soil conditions are critical in forecasting basin-scale rainfall-runoff relationships (3, 81–83). However, we highlight that across the Feather River basin (Fig. 7E), 10% drier soils produce comparable runoff relative to removing the snowpack entirely. In other words, a 10% soil moisture bias has the same impact as a 100% snowmelt bias in this case, underscoring the potency (and explanatory power) of soil moisture in runoff generation. This difference (33 mm) corresponds to about 0.34 km^3^, or 8% of Lake Oroville’s 4.36 km^3^ storage capacity. This consequence doubles with 25% drier soils. We note that the underestimates in event precipitation may explain the lower modeled streamflow in the Feather, as these biases share a similar magnitude (Fig. S13). In the Yuba and American, however, low-biased flows begin after most of the event precipitation with minimal modeled TWI, suggesting a model deficiency in baseflow.

These experiments demonstrate how increasingly efficient winter runoff volumes could be driven by saturated soils, and a somewhat “passive” snowpack that enables direct rainfall passage and contribution to runoff volumes despite several large, warm ARs. This mechanism is not unique to this study, as other cases of large, high-efficiency streamflow occur in other small, snow-dominated basins in the western United States—caused by wet soils and winter rainfall during periods of low evapotranspiration (84–86). Progressive soil saturation also augments streamflow responses to frequent mountain rainfall in the eastern United States (87), Canada (88), and Europe (89). The brief timescale (i.e., days) during which this mechanism operates implies that storm sequencing—especially over slow-draining soils with limited groundwater storage—can produce efficient floods, even in otherwise dry years (89). However, disentangling snowmelt and groundwater (i.e., baseflow) contributions from rainfall inputs in driving streamflow generation (84), and closing the water balance around these terms remains challenging in snow-dominated headwaters (90, 91). While baseflow may be linked to snowmelt in sandy catchments favoring infiltration (92, 93), parsing between them at the event scale requires fully coupled atmosphere-through-bedrock observation and modeling frameworks (94, 95). Nonetheless, we present evidence that the streamflow associated with ROS can be linked closely to the basin soil state as well as to the role of snowmelt, which we argue was smaller than prior research indicated. This link is crucial because it compels us to acknowledge that the exceptional “Oroville event,” hydrologically, was born from a chain of consecutive events that cumulatively primed the system (96) to respond to a single, high-impact event. Indeed, had a more widespread, “active” snowmelt response transpired during the 6F storm sequence, the risk of dam failure and catastrophic flooding would have arguably been much greater.

## Conclusions, Challenges, and Recommendations

Winter 2017 in California’s Sierra Nevada brought numerous landfalling warm ARs and multiple widespread flooding events. Two major storm sequences—beginning on 7 January and 6 February—had high snow levels and yielded extreme streamflow volumes in the Feather, Yuba, and American River basins (Fig. 1). Both storms shared several synoptic characteristics (Table S1), with the February event yielding less rainfall but more runoff than the January event. To explain this difference, we present evidence that snowmelt was not the primary flood driver, at least to the extent previously suspected. We show that (1) much of the snow cover on 24 January [underlying previous melt contribution estimates (19)] vanished prior to the event itself (Fig. 2), and (2) that hourly snow pillow responses to ROS revealed a potential to misinterpret SWE loss for snowmelt, as the energy to explain such losses was not always available (Fig. 5). Rather, a “passive” response to ROS may involve snow liquid water content rising to saturation and then draining, producing measured SWE gains and losses (Fig. 4). A series of idealized model experiments supported this interpretation suggesting that snowmelt during ROS was a relatively small part of a broader cause of the extreme runoff in the February event. The cascade of prior storm inputs gradually raised antecedent soil moisture and, in turn, led to increasingly efficient runoff (Fig. 6). Importantly, we show that event-scale ROS runoff generation is dramatically more sensitive to pre-event soil moisture than to event snowmelt (Fig. 7). This characteristic links successive storm events together and enhances responsiveness to a single, high-impact storm.

Nonetheless, the presence and danger of “active” snowmelt during mid-winter ROS as a potential flood driver should not be dismissed, and this is not our intention. Rather, we encourage developing an understanding of whether a snowpack will be “active” or “passive,” and how landscape saturation levels modify the associated flood risk during ROS. This large-scale understanding must avoid fixating on a single component in explaining entire events. The Oroville event itself was punctuated by spillway failures that exacerbated an extreme flood threat (53) despite the ROS component falling within a global, climatological range of snowmelt contributions to TWI (12). It stands alongside several historic flood events—across the Sierra Nevada (4), North America (17, 25), and worldwide (18)—that place a memorable spotlight on the challenges ROS presents to large, vulnerable systems.

Socially, we tend to remember past extreme events—our perceptions of which affect how we prepare for and respond to future events (97). Thus, an accurate, transferrable understanding and representation of the physical mechanisms of ROS will enable past events to better guide management responses in the future. However, the fact that we can posit a viable “alternative hydrology” of the Oroville event reveals an ambiguous perception of a past event. This “equifinality” presents a risk in choosing the most appropriate monitoring and/or modeling investments. For instance, what hydrologic compartment yields the most predictive benefit if monitored and/or assimilated at the watershed scale? The answer requires tangible field and/or model development efforts based on a working theory. However, our alternative explanation highlights conflicting priorities on which observations (e.g., soil versus snowpack) may best enhance future forecast skill. In preparing for a more uncertain future, this “gap” demands elevated observational and modeling capacities to identify the correct physical processes and their coupling to interpret such events. We recommend future efforts focused on the following:

1. Precipitation phase and intensity. This is a foundational yet elusive forcing in mountain environments, and a first-order control on deciphering the relative importance of precipitation versus snowmelt during ROS. Despite improvements in humidity-aware proxies (51) and the utility in vertically-oriented radars, the optimal approach in estimating precipitation phase is direct observation (98, 99). This may be partial to daylight hours and unclear during mixed-phase precipitation. However, combining such citizen science with denser, robust observation networks will bolster on-the-ground representation, and may help to better constrain weather model physics (70) and satellite retrievals of mountain precipitation.
2. Snowpack structure and energy balance. This largely dictates the volume and timing of the flood response to ROS. Snow pit observations provide snowpack stratigraphy, cold content, and liquid water content, helping to identify likely flow regimes and melt response to meteorological inputs. However, bracketing snow pit observations around ROS events requires intensive field campaigns. Dye tracer experiments carry similar benefits, but the post-hoc nature of these approaches is impractical for hazard and water supply monitoring. We recommend cost-effective automated or semiautomated efforts to map (100, 101) and to continuously and noninvasively monitor (102, 103) these quantities across watersheds (104) and land cover types. Process-scale monitoring of basic snow properties—which must be coupled with accurate surface and boundary layer characteristics—can be exploited for more representative modeling frameworks. Such measurements can improve process-aware constraints on the simplifying parameterizations that accompany models. They may also support developing more effective discretization schemes that respect the physical differences between matrix and preferential flow (33, 105). This is an important feature for next-generation models to conceptualize, given how dramatic these differences can be (22, 45).
3. Soil moisture. Landscape saturation strongly modulates ROS event runoff, despite a snowpack’s capacity to bypass soils and generate runoff directly (31, 45). The natural mitigation (or amplification) of runoff from modestly drier (wetter) soils (106)—even from high-impact ROS—implies an important constraint on the efficacy of floodwater-derived adaptive measures to counter long-term drought such as managed aquifer recharge and forecast-informed reservoir operations. Our ability to record and transmit soil moisture measurements in remote locations has improved greatly over time, significantly expanding soil moisture observation networks (49). However, the exact configuration of these networks that maximizes the benefit to mountain flood forecasting remains unclear. Accurate and coherently distributed soil moisture monitoring and data assimilation are necessary, although strong spatial and elevational variations in soil moisture (and SWE)—especially during storms—inherently limit point observations. Remote sensing [e.g., via *P*-Band signals of opportunity (107)] may provide concurrent measurements of root-zone soil moisture and SWE or snow depth at scales ∼1 km. Incorporating such information would be very useful in dissecting (and ultimately predicting) the hydrologic evolution of floods such as the Oroville event.
4. Graduation to scale. How the above-mentioned processes translate from the point and hillslope to basin scale is crucial to guide management decisions. Some of our analyses rely on observations located in flat clearings (and satellite-derived fSCA, which is partial to clearings and sparse vegetation). The snow pillow network occupies elevations as low as ∼1,600 m in the Sierra Nevada, roughly 300 m higher than the pre-6F event snowline (Figs. 1B and 2C). Lower-elevation ephemeral snow cover, while unmonitored, is likely a more “active” snowmelt source during ROS. While thinner, the areal extent and low cold content of such snowpacks become important in favorable storm sequences (e.g., warm, intense precipitation immediately following snowfall). In-storm shifts in this boundary and its SWE affect the tributary area and volume of ROS response (4, 108) and therefore should be tracked to understand its relationship to basin flood response. Moreover, being able to monitor exchanges between ground and surface water stores would help to evaluate the interrelationship between ROS, ephemeral snow cover, soil moisture, and runoff response as an integrated system. This monitoring effort may benefit from synthesized critical zone observations (92,109) and isotopic analyses (14) across landscapes. In an operational setting, these efforts converge (1) implicitly on improved model architecture that balances expedient forecasts with appropriate physical representation to yield skillful predictions, and (2) explicitly on improved quantitative precipitation and freezing level forecasts (110, 111) as well as initial model states (e.g., soil moisture and snowpack) used as input to hydrologic models.

Facing a climate more prone to high-impact ROS (1, 11), even as ROS events themselves become less frequent with snowpack declines (1, 11, 112), transdisciplinary efforts aimed toward understanding hydrologic connectivity across scales are paramount to overcoming these barriers. In addition to better understanding the governing processes of snowpack flow routing and snowmelt, we emphasize it is also worth looking up, down, and backward—“up” to understand within-storm changes to precipitation phase and boundary layer dynamics; “down” to understand the subsurface role in surface-groundwater exchange and basin-scale runoff generation; and “backward” to consider how soil and snow’s “memory” of preceding hydrometeorological events may affect subsequent ones. Such efforts will strengthen the operational tools (113) for managing water availability and hazards posed by ROS in a society dependent on increasingly warmer and variable winter precipitation.

## Material and Methods

### In-situ snow, soil, and meteorological measurements

Point measurements for SWE, snow depth, soil moisture, air temperature, wind speed, relatively humidity, and precipitation were obtained at an hourly timescale (or subhourly, if available) from multiple networks in the northern Sierra Nevada. All data were converted to UTC and metric units.

#### Snow water equivalent and snow depth

The California DWR manages a network of ∼130 automated monitoring stations across the Sierra Nevada that measure SWE from snow pillows. Some stations are run by the Natural Resources Conservation Service (NRCS) as part of the SNOTEL network—but all SWE data are posted to the DWR California Data Exchange Center (CDEC, http://cdec.water.ca.gov/snow/current/snow/index.html). We obtained hourly SWE from 22 snow pillows in the Feather, Yuba, and American River basins from CDEC (Table S2).

Several stations include ultrasonic snow depth measurements from either DWR or the American River Hydrologic Observatory [ARHO (114)]—a distributed sensor network with each station comprising a cluster of sensor nodes. We use the cluster median for four ARHO stations and seven DWR stations (Table S2). Both SWE and snow depth measurements were quality-controlled manually (the procedure is described in the Supplementary Material).

#### Soil moisture

Soil volumetric water content (VWC) is measured at few NRCS and DWR stations in our study basins (*n* = 1). To raise the number of samples, we obtained VWC measurements from other networks, including the Western Regional Climate Center (WRCC, https://wrcc.dri.edu/), the National Oceanic and Atmospheric Administration Physical Sciences Laboratory (NOAA PSL, https://psl.noaa.gov/data/obs/datadisplay/), and the ARHO. VWC values from NOAA PSL were converted from raw reflectometry measurements using the standard coefficients in the corresponding data logger manual (Table 4 in https://psl.noaa.gov/data/obs/instruments/SoilWaterContent.pdf). VWC values from ARHO are the cluster median at each station. Our expanded sample (*n* = 7) occupies an elevation range from ∼1,050 to 2,700 m. The depth and timestep of data vary by station and network (Table S3). We aggregated subhourly measurements to hourly timesteps after quality control via screening measurements when soil temperatures dropped below 0ºC.

VWC served three purposes in this study. First, the lowest-elevation sensor was used to infer ephemeral snowmelt between the 7J and 6F events. Second, we used the shallowest (5 to 10 cm) available sensors with collocated SWE to infer “passive” snowpack behavior as SWE increasing simultaneously with VWC during rainfall. Using shallow sensors minimized the effect of different soil hydraulic properties on the timing between SWE and VWC changes during ROS. Third, we use the elevation gradient in VWC measurements to show a widespread increase in antecedent conditions resulting from winter storm events.

#### Surface meteorology

Precipitation, air temperature, relative humidity, and wind speed measurements were obtained from CDEC, WRCC, and MesoWest. The MesoWest portal (https://mesowest.utah.edu/) hosts data from the National Weather Service and other Remote Automatic Weather Stations. We screened available measurements for each variable and applied quality control prior to analysis (described in detail in the Supplementary Material). We used a total of 31 precipitation gauges to bound the range of precipitation during each storm (Table S4). We used a total of 41 stations reporting temperature, humidity, and wind speed, although not all measurements were suitable for both storms of interest (Table S4; Supplementary Material). Temperatures and winds were summarized for each storm at four elevation bands (Table S1).

### Streamflow

Stream discharge measurements were obtained from the USGS National Water Information System (https://waterdata.usgs.gov/nwis). We used a total of nine gauges in this study (Table S5), but only four report subdaily (15-minute) measurements. These higher-frequency measurements were used in analyses with other subdaily data. Daily measurements were used to illustrate how streamflow evolved over the winter season. We selected gauges from the Geospatial Attributes for Gauges for Evaluating Streamflow (GAGES-II) data set (115) that reflect unimpaired streamflow—either by its GAGES-II classification as “Reference,” or by removing stations below reservoirs or diversions. We visually inspected observations to remove candidate gauges affected by upstream regulation (e.g., as “stepwise” changes unassociated with precipitation or snowmelt) or otherwise missing/erroneous measurements.

We also obtained daily full-natural flow at Oroville Dam—draining the entire Feather River basin—from CDEC (station code ORO).

### Snow level radars

Several frequency-modulated continuous wave snow level radars managed by NOAA PSL occupy the Central Valley and foothills of the Sierra Nevada. The BBH from these upward-looking *S*-band (2.8 to 3.0 GHz) radars estimate the melting level aloft, derived from an algorithm that inspects range gates for the maximum reflectivity and increasing Doppler fall velocity associated with melting snowfall (116). The algorithm involves a self-consistency test with neighboring 30-second measurements as a quality control measure for the aggregated 10-minute measurements. We used 10-minute BBH measurements from the Oroville and Colfax radars in this study (Table S6) as a measure of the likely phase of precipitation. We note that because these sensors are located in the Central Valley, they may not always truly reflect mountain based melting levels (80).

### Satellite remote sensing

True-color images from NASA Worldview (https://worldview.earthdata.nasa.gov/) supported qualitative assessment of cloud and snow coverage. We obtained estimates of fSCA from the MODIS Snow-Covered Area and Grain Size (MODSCAG) algorithm (117), which retrieves these properties daily at 500 m. Scenes were used for near-cloudless (below 20%) days that had no apparent cloud coverage in Worldview. We then used fSCA to calculate the regional snowline elevation over the aggregated study basins (118).

### Atmospheric reanalysis

The 5th generation of atmospheric reanalysis from the European Center for Medium-Range Weather Forecasts (ERA5) provides hourly atmospheric variables on a 0.25º grid (119). We obtained ERA5 geopotential, air temperature, specific humidity, and zonal and meridional winds at 27 pressure levels (from 1,000 to 100 hPa) from the Copernicus Climate Change Service’s Climate Data Store (https://cds.climate.copernicus.eu/). We also obtained hourly 0.1º surface wind and 0ºC altitude variables from ERA5-Land (120).

### Synoptic analysis

To assess synoptic differences between the January and February storm events over the study basins, we calculated the MSE at each pressure level and the integrated vapor and heat transports (IVT, IHT). Equations are presented in the Supplementary Material. In essence, IVT and IHT are wind-weighted quantities of moisture and heat movement, respectively, which are the key ingredients to turbulent (latent and sensible, respectively) heat fluxes at the surface, depending on the moisture and heat contents of the snowpack surface. We took the difference in MSE between 500 and 850 hPa as a relative measure of static instability (59), where smaller gradients indicate less static stability and thereby a greater uplift tendency and conductance for turbulent fluxes. Taken together, these metrics represent the relative strength of AR-related snowmelt drivers (17).

To capture the prevailing conditions during rainfall, we considered the above metrics during hours when BBH (at either Oroville or Colfax) exceeded 1,600 m. This threshold nominally represents the lower regions of the snow pillow network (Table S2) to suggest that rainfall is likely occurring over low-lying snow cover, at the very least. Given that this value resided on average a few hundred meters above the regional snowline elevation, this threshold inherently accounts for the regional lowering of upwind snow levels (80) that can positively bias BBH values applied downwind for precipitation phase partitioning. Both IVT and IHT were expressed as accumulations (kg m^−1^ and J m^−1^) over the high-BBH timesteps, while values for the MSE gradients were averaged.

### Cumulative discharge and rainfall comparisons

To assess both runoff efficiency and the notion of snowmelt augmenting TWI above rainfall alone, we compared rainfall estimates to observed discharge at each subdaily USGS gauge over four intervals in the snow season. We hypothesized that runoff efficiency would grow over the course of the winter as TWI accumulated to raise antecedent soil moisture, and that the presence of snowmelt and rainfall together would bring discharge above rainfall totals.

We partitioned gridded (4-km), 6-hourly precipitation from the California Nevada River Forecast Center (CNRFC, https://www.cnrfc.noaa.gov/arc_search.php) over the drainage areas of each subdaily USGS gauge using the nearest BBH measurements. We first aggregated the 10-minute BBHs to hourly values. We filled the remaining gaps in the hourly time series using ordinary least squares regression of hourly BBH against the 0ºC altitude from the nearest ERA5-Land pixel from November 2016 through early May 2017. Regression results at the Oroville (*n* = 664) and Colfax radar (*n* = 671) yielded *R*^2^-values of 0.93 and 0.95, respectively, with a standard error of 0.01 m. This gap-filled time series was then aggregated to 6-hourly values to match CNRFC, then lowered by 200, 400, and 600 m to test different degrees of snow level bending (80). We considered rainfall as CNRFC precipitation at pixels below the aggregated snow level, and compared rainfall and observed streamflow accumulations for the following periods: (1) early winter—from 15 November (the first large rainfall event) through December, (2) January—encompassing the 7J event, (3) February—encompassing the 6F event, and (4) early spring—from 16 March (the first rainfall event) through early May. These time frames were chosen such that rainfall began early in the period and ceased before the end of the period, but allowing several days of concentration time before the next rainfall event.

### VIC model experiments

We used the spatially distributed VIC (version 4.2d) hydrologic model (61) to experiment and estimate the energy balance during ROS. The VIC snow model (38) simulates mass and energy transfers between the atmosphere, overlying vegetation, and underlying snowpack, including drip, interception, and sublimation processes. The snow model has been validated in the Sierra Nevada and utilized previously to investigate ROS flooding across the United States (1, 3). We ran VIC here in energy balance mode, with grid cells subdivided into five elevation bands and further into up to 12 vegetation tiles. Energy and mass states and fluxes are computed for each grid cell subdivision and output as the area-weighted average. Daily, 1/16º (∼6-km) forcings of total precipitation, wind speed, and minimum and maximum air temperature were obtained from an extended version of the Livneh et al. (121) product (L15), which adjusts its temperature and precipitation fields for orographic effects using the Parameter-elevation Regressions on Independent Slopes Model (122) climatology. The mountain microclimate simulation model (123) derives the remaining forcings used by VIC—downwelling short- and long-wave radiation, and vapor and atmospheric pressure. It permits subdaily simulation by disaggregating the daily forcings to reflect diurnal variation. Precipitation was partitioned into liquid, solid, and mixed phases by an air temperature range in which precipitation falling below −0.5 and above +0.5ºC were classified as snowfall and rainfall, respectively, with mixed rain and snow calculated by interpolating between temperatures within this range. We also explored a temperature range of 0.0 to +2.0ºC, which does not affect results. We used the L15 land surface parameters, which were calibrated for major river basins (121) and applied successfully in several studies (1, 3, 93, 112) across the United States. We ran this configuration at an hourly timescale over the study basins beginning 1 October 2015 to allow 1 year of spin-up time. However, we use 3-hourly output to compromise needs for subdaily variation while avoiding artifacts in the disaggregation of daily forcing data

VIC simulations served three purposes in this study. First, we approximated net energy inputs at grid cells nearest to snow pillows to determine whether the observed SWE decreases in “pulses” during ROS could be explained as snowmelt. To better represent the exposure at snow pillows, turbulent heat fluxes were calculated as in Andreadis et al. (38), using modeled snow depth and a snow surface roughness length of 1 mm (16) to estimate aerodynamic resistance. Net-positive (downward) fluxes of net radiation, turbulent fluxes, and rainfall heat advection when modeled snow surface temperatures were above −0.5ºC were integrated over the durations of observed SWE decreases during the 7J and 6F events. Net fluxes were converted to equivalent ice melt rates by dividing the energy values by values for water density and latent heat of fusion. These were compared to the sum of negative changes in snow pillow SWE. Instances where modeled melt had met or exceeded observed SWE losses were deemed to have sufficient energy inputs to completely explain losses as snowmelt, as opposed to “passive” routing of liquid through snow.

Second, we conducted a no-snow experiment to approximate the role of snowpack in augmenting the 6F event flood response. We set all snow variables in the model state (SWE, fSCA, liquid water content, snow density, cold content, and pack and surface temperatures) to 0 (mm or ºC) on 5 February and reinitialized the model with liquid-only precipitation (which was equivalent to the liquid precipitation in the baseline simulation). Snow/rain discrimination temperatures were set to −273ºC to ensure all precipitation fell as rain (rather than raising the forcing air temperature, which may affect evapotranspiration). The no-snow scenario effectively simulates a completely passive snowpack response to the 6F event, in which preferential flow is the primary flood mechanism. This provides a benchmark for describing the role of an active snowpack in the event and inherently accounts for any flood-reducing capacity of deeper, colder snow. We report the difference in total event TWI between the no-snow and baseline scenarios to (1) isolate the snowpack role in the event, and (2) to avoid differing rainfall estimates from confounding the comparison of snowpack augmenting TWI across studies (19).

Lastly, we simulated the 6F event with systematically drier soils to test our hypothesis of the event flood response being driven more by wet soils than by snowmelt. We reduced the soil moisture at each of the three soil layers in the 5 February state by scaling each layer by a given fraction (10, 25, 50, and 75%) before reinitializing the model with unperturbed snowpack and precipitation. In addition to the energy balance and snow pillow analysis, we report modeled inflows to the soil column (TWI), total runoff (combining surface runoff and baseflow), and soil moisture (normalized between grid cell VWC values for field capacity and wilting point).

## Supplementary Material

pgac295_Supplemental_File

## Data Availability

All data used in this study are publicly available. We have also deposited all data, code, and VIC simulations in Zenodo—a fair and compliant repository; https://doi.org/10.5281/zenodo.7103330–to make the work easily reproducible.
