# Supplementary material for: Watershed memory amplified the Oroville rain-on-snow flood of February 2017
Source: PNAS Nexus. 2022 Dec 16;2(1):pgac295. doi: 10.1093/pnasnexus/pgac295 (PMC9832955; doi:10.1093/pnasnexus/pgac295)
Supplement: pgac295_Supplemental_File [file pgac295_supplemental_file.docx]

**
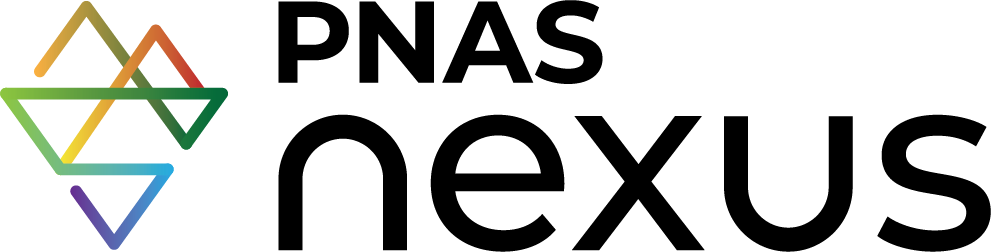
**

**Supplementary Information for**

Watershed memory amplified the Oroville rain-on-snow flood of February 2017

Kayden Haleakala* (1,2), W. Tyler Brandt (2), Benjamin J. Hatchett (3), Dongyue Li (4), Dennis P. Lettenmaier (4), Mekonnen Gebremichael (1)

(1) Department of Civil and Environmental Engineering, University of California Los Angeles, Los Angeles, California

(2) Center for Western Weather and Water Extremes, Scripps Institution of Oceanography, San Diego, California

(3) Division of Atmospheric Sciences, Desert Research Institute, Reno, Nevada

(4) Department of Geography, University of California Los Angeles, Los Angeles, California

*Corresponding author: Kayden Haleakala

Email: [khaleakala@g.ucla.edu](mailto:khaleakala@g.ucla.edu)

**This PDF file includes:**

Supplementary text

Figures S1 to S13

Tables S1 to S6

SI References

**Supplementary Information Text**

This text describes (1) the selection and quality control procedure for in situ meteorological and snow data, (2) the calculations pertaining to our synoptic-scale analyses, and (3) the experimental wind-correction of precipitation noted in the main text.

**Meteorological data collection and quality control**

Hourly (or sub-hourly, if available) measurements of precipitation, air temperature, relative humidity, and wind speed were obtained from a variety of observation networks. These include the California Data Exchange Center (CDEC, <http://cdec.water.ca.gov/snow/current/snow/index.html>), the Western Regional Climate Center (WRCC, <https://wrcc.dri.edu/>), MesoWest (<https://mesowest.utah.edu/>), and the American River Hydrologic Observatory (ARHO, ^1^). We employed the following screening and quality control routine for each variable.

Accumulated precipitation data were collected at native sampling intervals (ranging from 2 minutes to 1 hour). Missing values were imputed for 1-hour gaps or shorter by interpolating across bounding values. Longer gaps were ignored. Incremental precipitation was then calculated via first difference. High-elevation (above ~2,000 m) stations were commonly removed from analysis after visual inspection showed obvious wind effects and/or sticking (i.e., sparse precipitation increments during periods of heavy continuous precipitation at neighboring stations). Negative increments were set to zero, and increments above 25 mm were flagged as missing. Stations missing more than 10% of values during the storms of interest were removed from analysis. The remaining stations were compared to the nearest snow pillow and screened from analysis if total accumulation season (November through March) precipitation fell below 1 April 2017 snow water equivalent. This resulted in a total of 31 suitable gauges (Table S4).

Wind speed, air temperature, and relative humidity data were collected in a similar manner as described above. At temperatures near 0˚C, propeller anemometers can melt and re-freeze the snow that falls on them, stopping the sensor’s motion and resulting in an artificially “calm” period. We used a threshold of 6 hours beyond which winds speeds of 0 m s^-1^ winds were flagged as missing. Wind speeds above 20 m s^-1^ were flagged as missing. Air temperature measurements above 40˚C in absolute value were flagged as missing, while relative humidity values below (above) 0% (100%) were flagged. We interpolated across single-timestep gaps for both variables. We used the cluster medians for ARHO temperature and humidity measurements. Variables with more than 10% values missing during the storms of interest were excluded from analyses on the respective storm, resulting in a total of 41 stations (Table S4).

**Snow data collection and quality control**

Hourly snow water equivalent (SWE) and snow depth measurements (where and when available) were obtained from CDEC. We employed the following quality control routines for each variable.

For SWE, large negative values (less than -1 cm) were flagged as missing, as were erroneously high (approximately 4 m) values Smaller negative values (between -1 and 0 cm) were set to zero. We also flagged erroneous “spikes” in the hourly time series. These spikes are characterized by large (e.g., > 5 cm in absolute value) changes that deviate briefly from the surrounding neighborhood of values before returning. Spikes may occur over single timesteps, or values may jump to an erroneous series of values for several hours before returning to the typical neighborhood of values. Such increases in SWE are unassociated with precipitation, and decreases are highly unlikely to be midwinter snowmelt, as such a melt rate reflects an unrealistic energy requirement (e.g., the energy melting 15 mm hr^-1^ from an isothermal snowpack is equivalent to the solar constant – 1367 W m^-2^). We also manually screened for bridging effects on SWE measurements – frozen snow layers may prevent additional snowfall from being recorded by a snow pillow until the bridge breaks. This results in a stationary SWE time series accompanied by increasing snow depth, followed by an abrupt increase in SWE. We flagged bridging periods once SWE values began to display zero variance.

CDEC snow depth measurements were quality controlled in a similar manner, with high (exceeding 7 m) and low (less than -1 cm) values flagged as missing, and small negative (between -1 and 0 cm) values set to zero. Spikes were much more common in snow depth time series, sometimes such that typical versus erroneous neighborhoods of values were not visually distinguishable. If SWE measurements were available, we addressed this issue by flagging neighborhoods of values reflecting uncharacteristic (exceeding 600 kg m^-3^) bulk snow densities for melting seasonal snowpacks^2^. Snow depth measurements from ARHO (station IDs ALP, CAP, RBB, SCN, VVL in Table S2) were previously quality controlled and gap-filled (described in Malek et al.^3^). However, we re-introduced gaps lasting longer than 24 hours during storm periods (e.g., at RBB in 8-10 January 2017), to avoid the linear interpolation across such gaps misrepresenting the timing of changes in bulk snow density used to infer precipitation phase.

**Calculations supporting synoptic analysis**

We obtained hourly, 0.25˚ geopotential (Φ, m^2^ s^-2^ or J kg^-1^), air temperature ($T_{air}$, K), specific humidity ($q_{air}$, kg kg^-1^), and zonal (u, m s^-1^) and meridional (v, m s^-1^) winds at 27 pressure levels (from $P_{1}$=1,000 to $P_{2}$=100 hPa) from the 5^th^ generation of atmospheric reanalysis from the European Centre for Medium-Range Weather Forecasts (ERA5)^4^. Geopotential heights were obtained by dividing Φ by gravitation acceleration, g (9.81 m s^-2^). We used metrics derived from ERA5 variables to assess synoptic differences between the January and February storm events of interest. Over the study basins, we consider the following metrics:

First, the integrated vapor transport (IVT, kg m^-1^ s^-1^) was calculated as:

$$IVT=\sqrt{\left( \frac{1}{g}\int_{P_{1}}^{P_{2}} q_{air}u dP \right)^{2}+\left( \frac{1}{g}\int_{P_{1}}^{P_{2}} q_{air}v dP \right)^{2}}, Eq (1)$$

Second, integrated heat transport (IHT, J m^-1^ s^-1^) was calculated as:

$$IHT=\sqrt{\left( \frac{c_{P}}{g}\int_{P_{1}}^{P_{2}} T_{air}u dP \right)^{2}+\left( \frac{c_{P}}{g}\int_{P_{1}}^{P_{2}} T_{air}v dP \right)^{2}}, Eq \left( 2 \right),$$

where $c_{P}$ is the specific heat of dry air at constant pressure (1,005 J kg^-1^ K^-1^).

Lastly, moist static energy (MSE, J kg^-1^) was calculated at discrete pressure levels,

$$MSE=c_{P}\theta+L_{v}q_{air}+\Phi, Eq \left( 3 \right),$$

where $L_{v}$ is the latent heat of vaporization (assumed to be constant at 2.5x10^6^ J kg^-1^). The potential temperature, θ (K), was calculated as:

$$\theta=T_{air}\left( \frac{P}{P_{0}} \right)^{-\frac{R_{d}}{c_{P}}}, Eq \left( 4 \right),$$

where $P_{0}$ is 1,000 hPa and $R_{d}$ is the dry air gas constant (287 J kg^-1^ K^-1^).

**Testing precipitation undercatch as an artifact of snowmelt augmenting precipitation**

We argue that the degree of snowmelt contributing to the exceptional runoff from the February storm event was lower than previously reported. A prior study quantified the relative increase in terrestrial water input (TWI) during rain-on-snow from snowmelt as the ratio of snowmelt to rainfall^5^. They used 4-km, 6-hourly precipitation from the California Nevada River Forecast Center (CNRFC), which is a topographically-corrected mosaic of gauge observations. While gauge undercatch can occur in exposed, windy areas, the CNRFC product does not apply a wind correction. Any wind-induced biases in the product would be baked into this estimate of snowmelt augmenting TWI and can conceivably inflate it. We considered this possibility as part of our argument by using hourly ERA5-Land^6^ wind vectors to correct CNRFC precipitation. We resampled CNRFC to hourly values with uniform scaling and mapped the 0.1˚ ERA5-Land wind vectors to the CNRFC grid via nearest neighbor. We applied a simple correction scheme from Masuda et al. ^7^ assuming a gauge height of 2 m, anemometer height of 10 m, and the correction coefficient for a heated tipping bucket gauge experiencing rainfall (their Table 2 and Equations 1 and 2). For both the January and February events over the Feather River basin, we report the ratio of corrected- to accumulated precipitation totals for various roughness lengths. It should be noted that increases to wind-corrected precipitation is likely low because (1) reanalyzed winds can underestimate true wind patterns in mountainous areas from smoothing over complex terrain, and (2) the highest elevations in the basins may have experienced snowfall, which would require a more aggressive correction^7^.

**
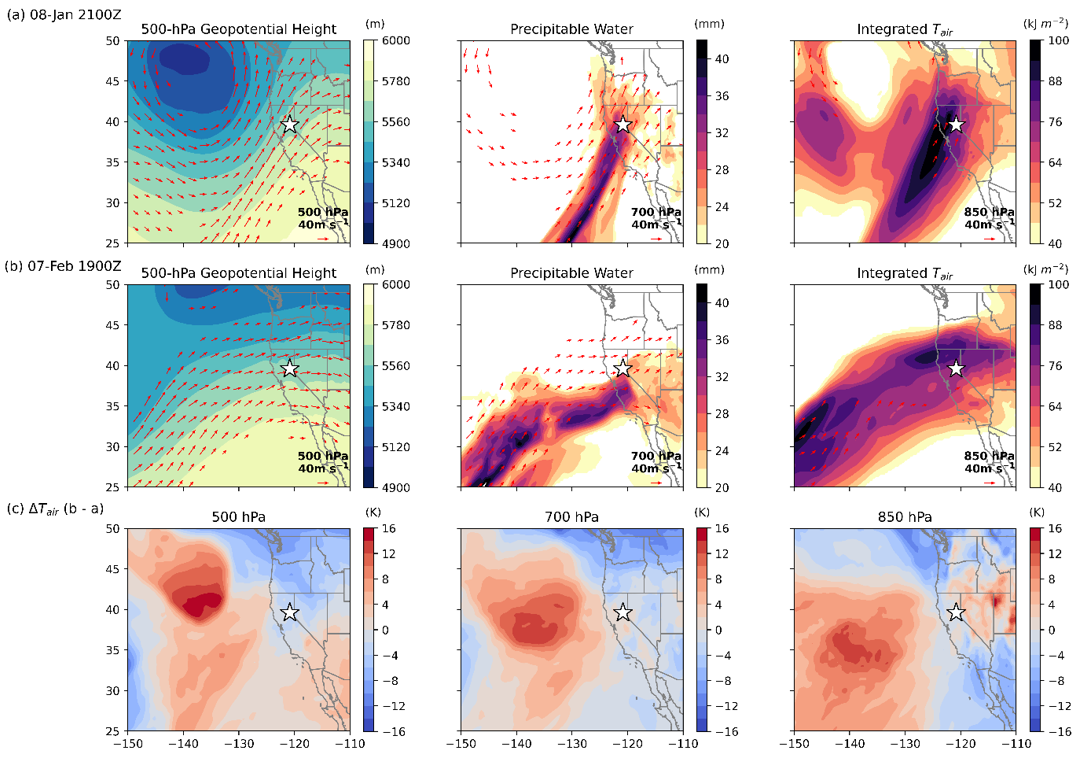
Fig. S1.** ERA5 500-hPa geopotential height, and column-integrated water vapor and heat at mid-storm timesteps in the (top row) January and (middle row) February storm events. Timesteps resemble moments in each storm when rain-on-snow elicited snow pillow responses. Wind vectors above 20 m s^-1^ are shown at 500, 700, and 850 hPa, which demonstrate stronger winds during the January event. In-storm air temperature differences at these pressure levels (bottom row) showcase the oncoming cold front at the end of the January event.


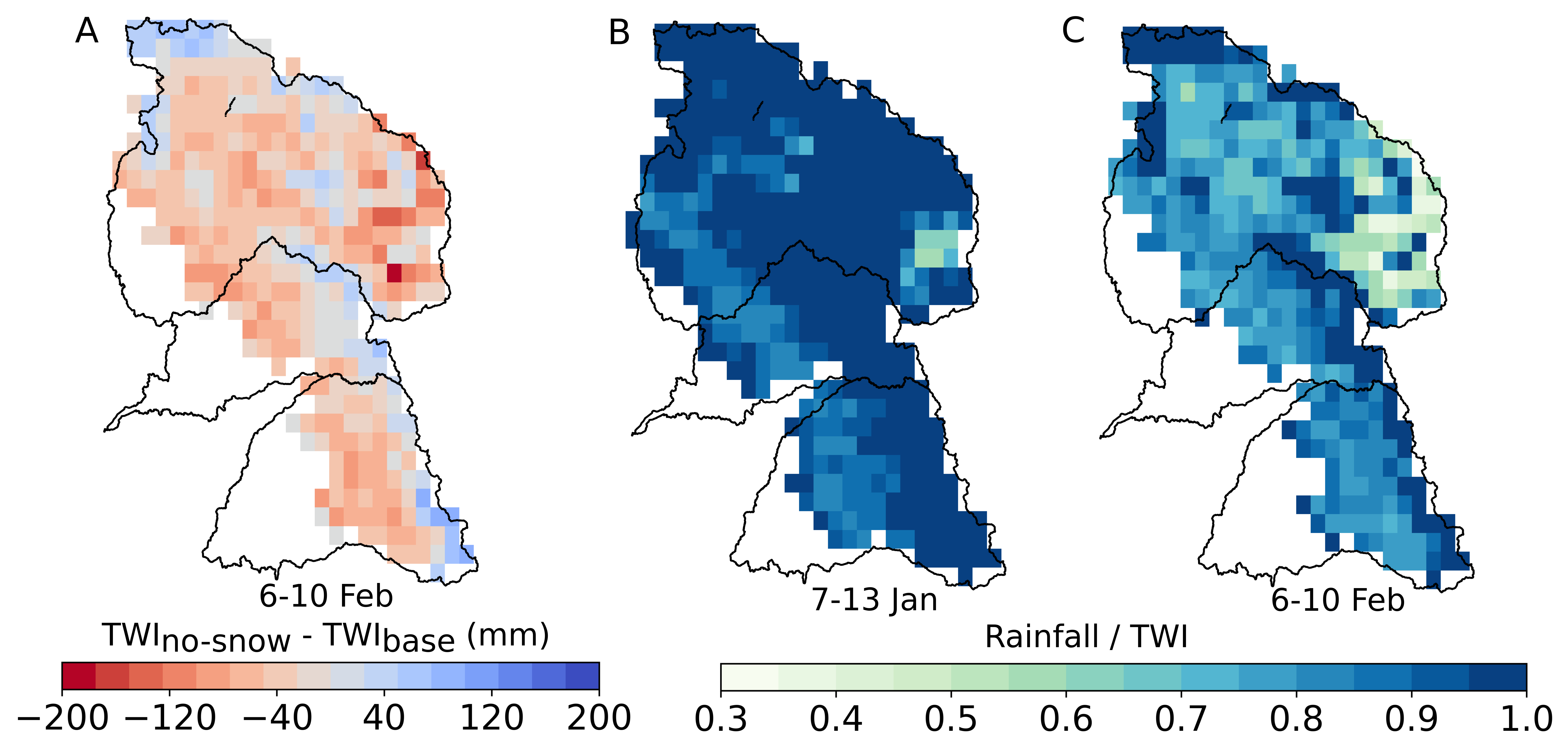


**Fig. S2.** Same as manuscript Fig 3B-D, exploring a warmer precipitation partitioning scheme (0 to 2 ⁰C as opposed to -0.5 to +0.5 ⁰C). Modeled differences in the 6-February ROS event-accumulated TWI between baseline and no-snow scenarios (A) represent the role of snowpack in affecting TWI (negative values). Accumulated rainfall to TWI ratios over the (B) 7-January event and (C) 6-February event show a dominant rainfall contribution to TWI. Results are broadly insensitive to precipitation partitioning.


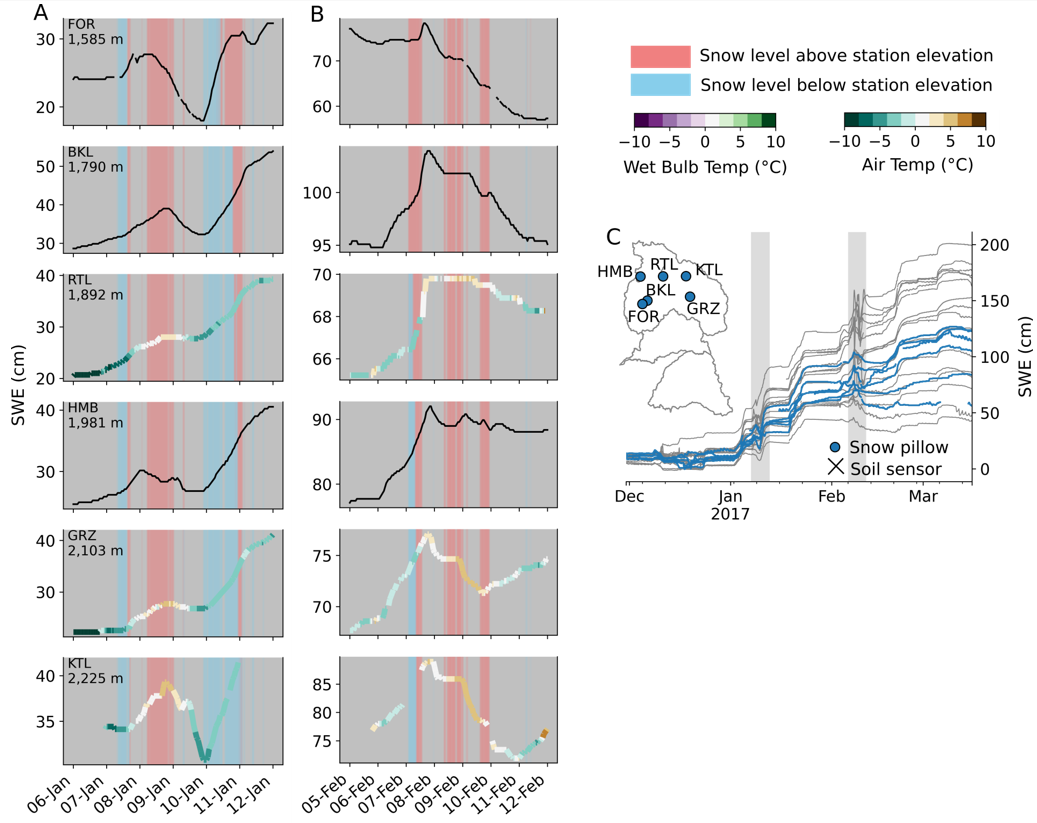


**Fig. S3.** Same as manuscript Fig 4A-C, for a set of snow pillows without snow depth measurements in the Feather River basin. Black lines indicate no available air temperature measurements.


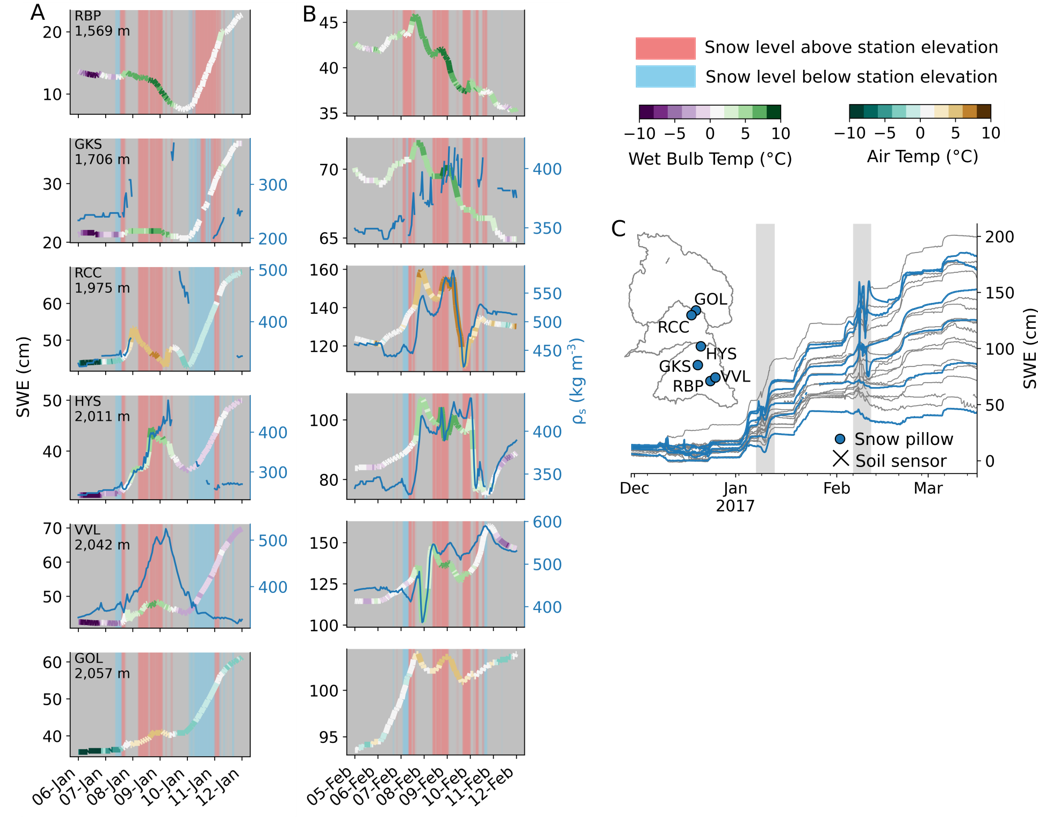


**Fig. S4.** Same as manuscript Fig 4A-C, for a set of snow pillows in the Yuba and American River basins.


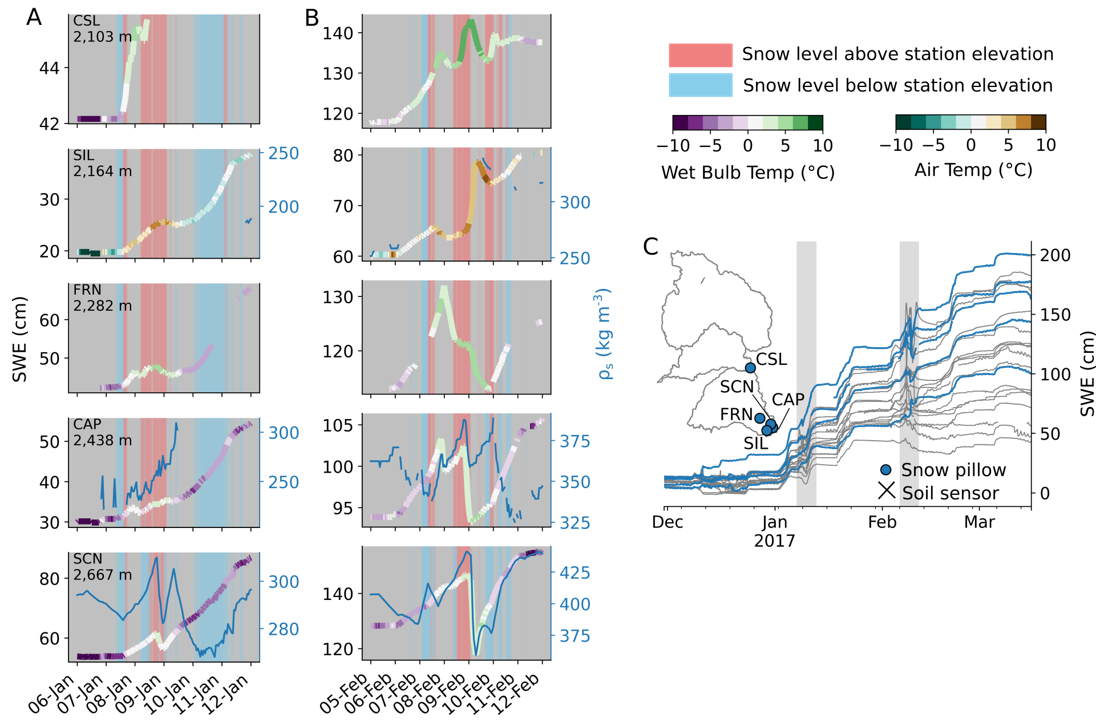


**Fig. S5.** Same as manuscript Fig 4A-C, for the remaining snow pillows not shown in Fig S3-S4.


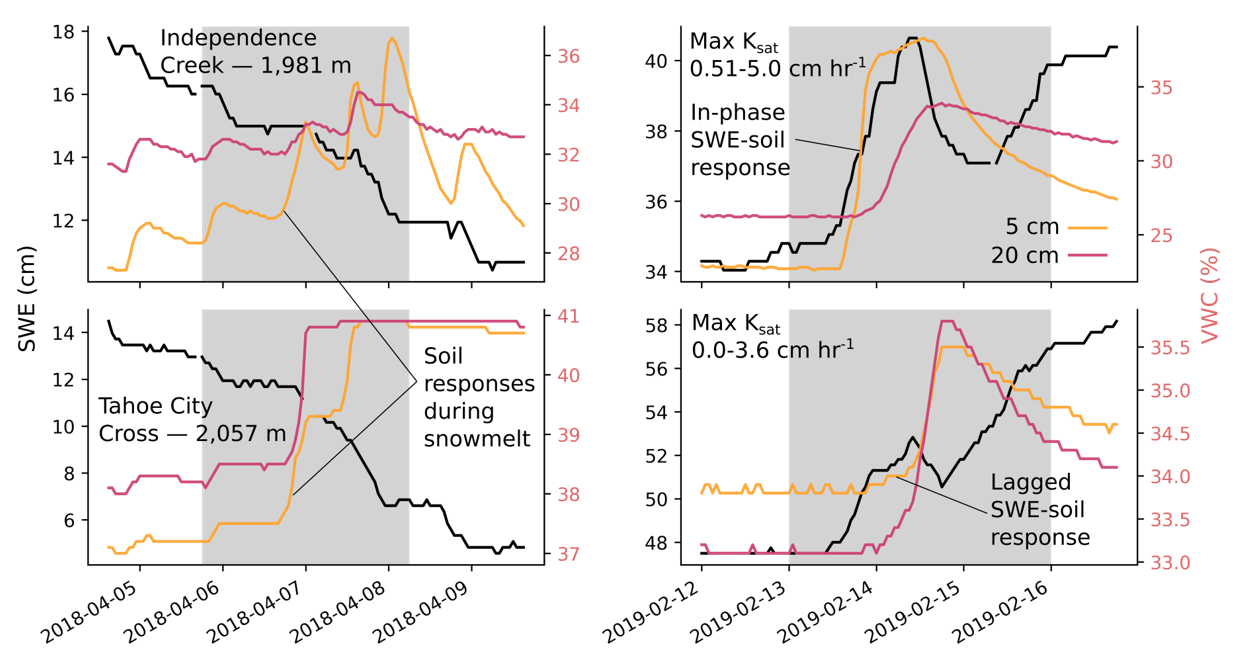


**Fig. S6.** Counterexamples to the interpretation that in-phase SWE-soil moisture responses to ROS indicate rainfall passively routing through the snowpack. Shown are SWE and soil moisture time series from a ROS event in the spring of 2018^8^ and winter of 2019^9^ from two snowpack telemetry stations managed by the Natural Resource Conservation Service (Independence Creek, SNOTEL ID 540 and Tahoe City Cross, SNOTEL ID 809; https://wcc.sc.egov.usda.gov/reportGenerator/) . They are both located lee of the Sierra Nevada crest (outside our study basins) in the Truckee basin and rest at similar elevations. The spring 2018 event (left column) occurred during the snowmelt season, where soil moisture increased coincidentally with SWE decreases. The winter 2019 event (right column) resulted in similar “pulses” in SWE with associated increases in soil moisture as reported in the manuscript. However, the Tahoe City Cross station soil moisture response appears out-of-phase with the SWE response, compared to the in-phase response recorded at Independence Creek. This may be a matter of strong differences in saturated hydraulic conductivity, as lower-conductivity soils will respond later to the same surface input at the same depth. Provided that this out-of-phase relationship is similar to the soil’s response to “active” snowmelt, it is inappropriate to infer passive ROS responses from this behavior alone. Maximum saturated hydraulic conductivity values were obtained from the National Cooperative Soil Survey for the station location’s predominant soil type (https://websoilsurvey.nrcs.usda.gov).

**
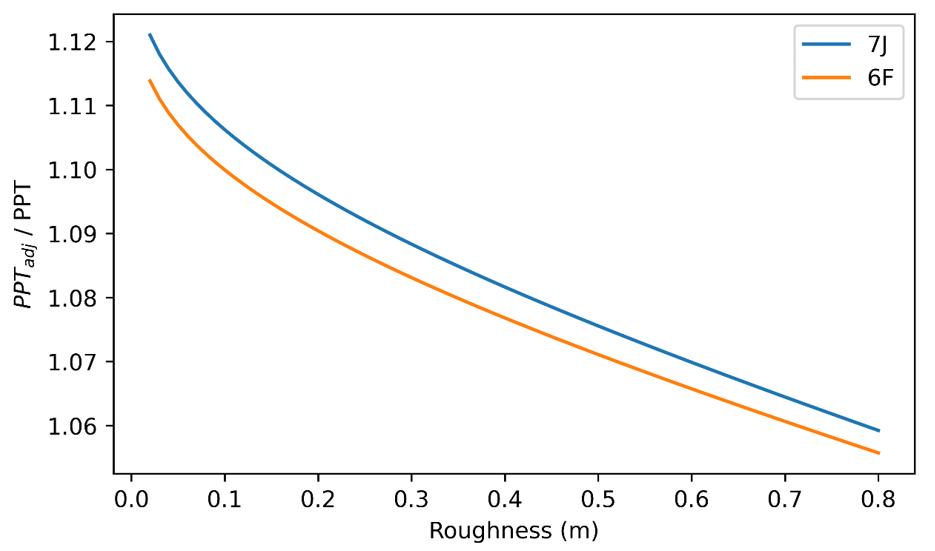
**

**Fig. S7.** The ratio of wind-corrected to raw CNRFC precipitation over the Feather River basin during the 7 January and 6 February storm events at various roughness lengths. Wind correction raises event-total approximately 6 to 12 percent, depending on the land cover.


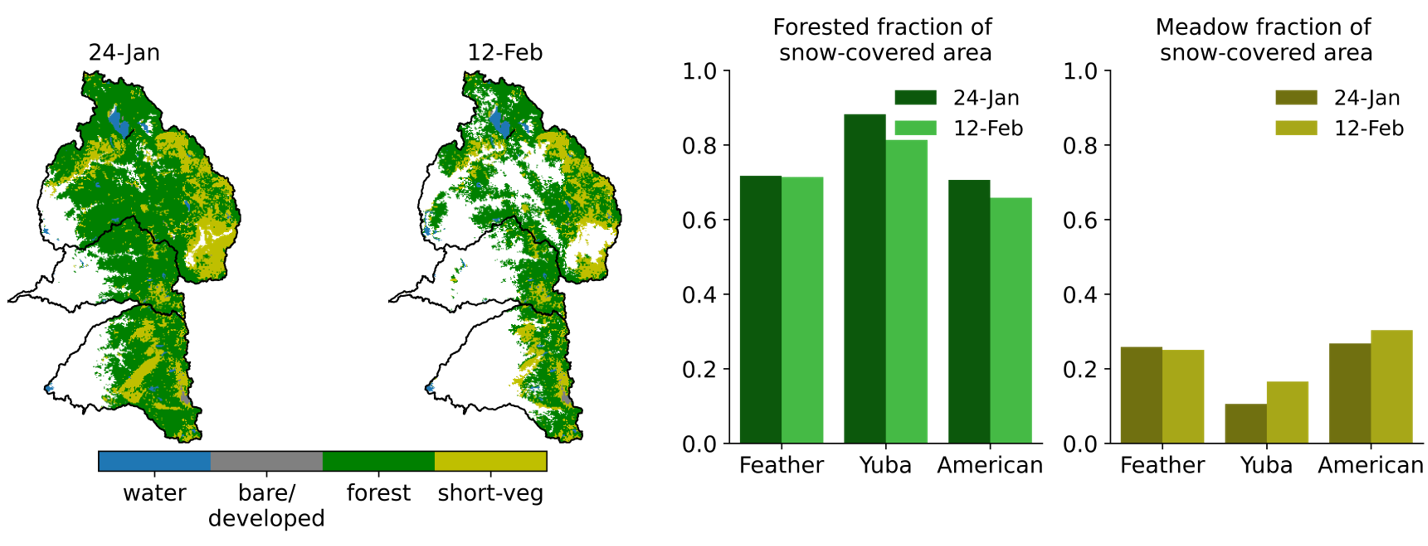


**Fig. S8.** Land cover classifications over snow-covered areas before and after the 6 February ROS event show the study basins’ snow-covered areas are heavily forested. Classification was determined from the 2016 National Land Cover Database (NLCD; https://www.mrlc.gov/) – the 30-m array was upscaled to the MODIS 500-m grid using the most frequently occurring class in the larger grid cell.


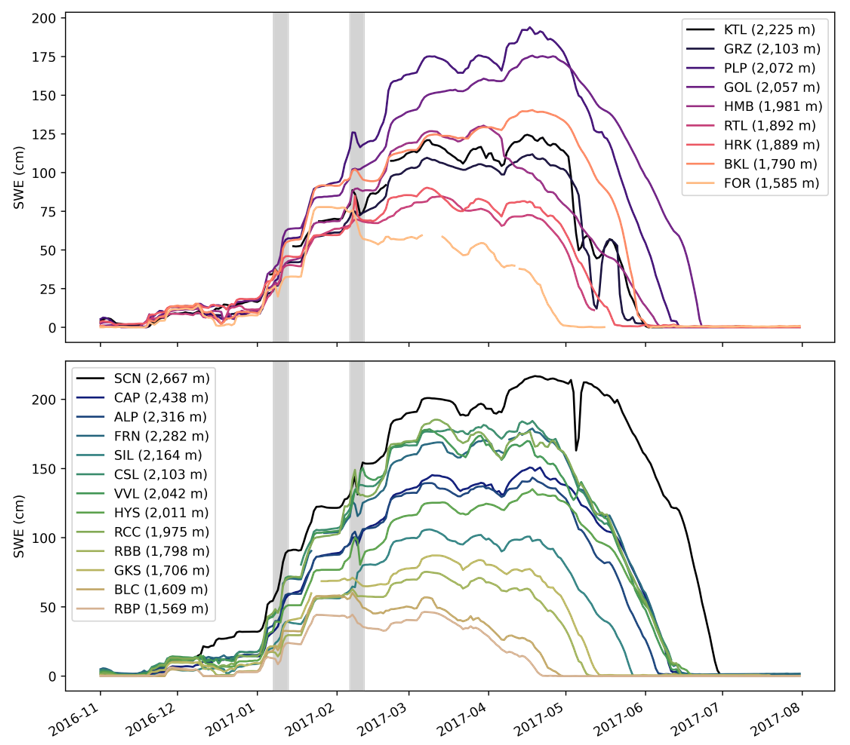


**Fig. S9.** Daily SWE in water year 2017 – peak SWE occurs in March-April at most snow pillows.


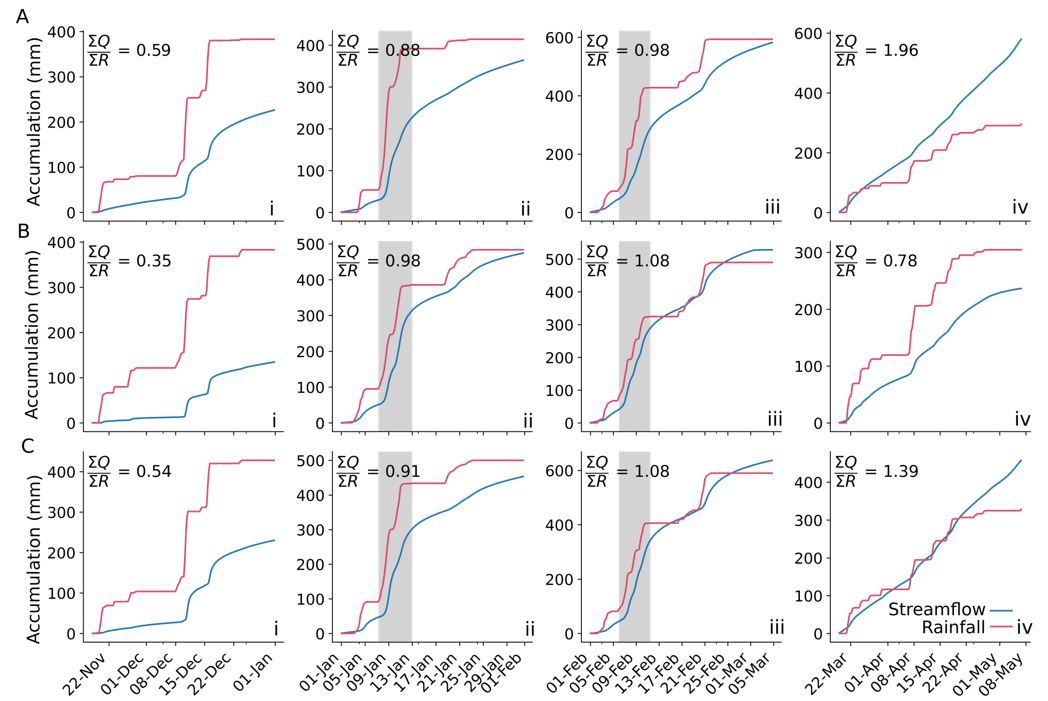


**Fig. S10.** Streamflow and estimated rainfall (the liquid proportion of total precipitation) accumulations at stream gages in (A) the northern Yuba River (USGS gage 11413000), (B) southern Yuba River (USGS gage 11418500), and (C) the North Fork American River (USGS gage 11427000) for the same periods as in manuscript Fig 5D-G. Rainfall values are aggregated from gridded precipitation over the catchment area upstream of the gage.


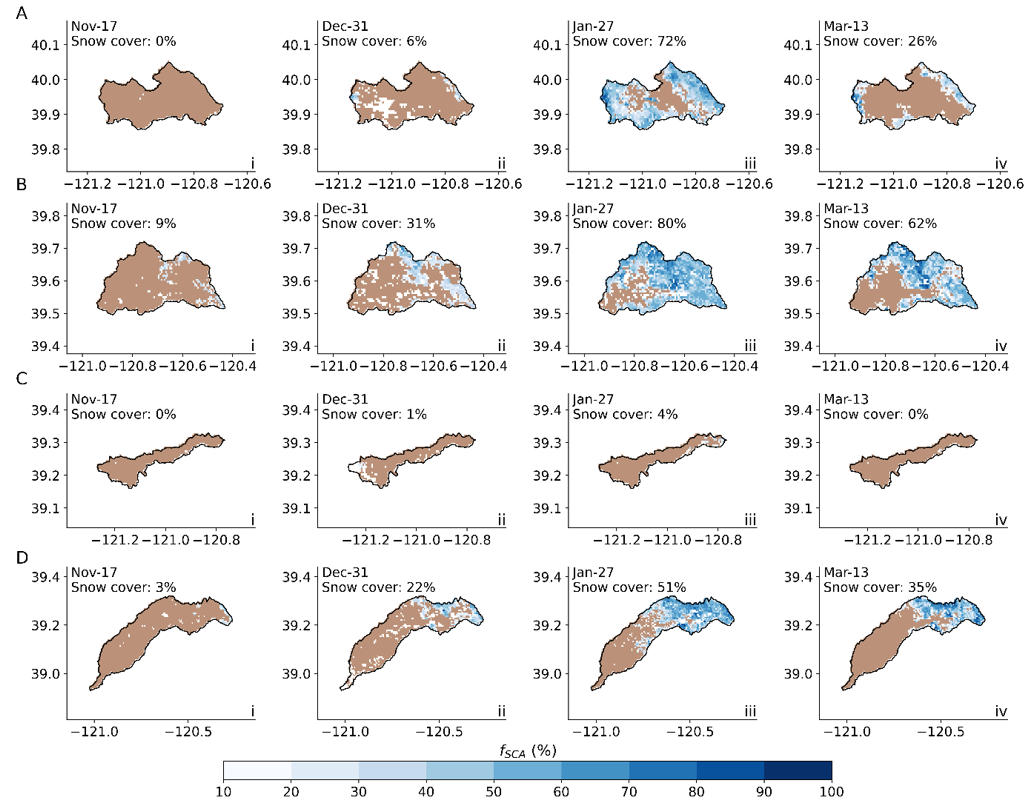


**Fig. S11.** Antecedent snow cover of basin areas on clear days preceding the time periods shown in Fig 5D-G in the manuscript and Fig S10 above. Drainage areas pertain to USGS gauges in the (A) central Feather, (B) northern Yuba, (C) southern Yuba, and (D) American for (i) early-season, (ii) January – preceding the 7J event, (iii) February – preceding the 6F event, and (iv) spring periods. Maps show MODIS fractional snow-covered area (fSCA, blue), masking snow-free regions (fSCA < 10%, brown). Annotated snow cover percentages reflect the proportion of catchment area that is snow covered (fSCA $\geq$10%).


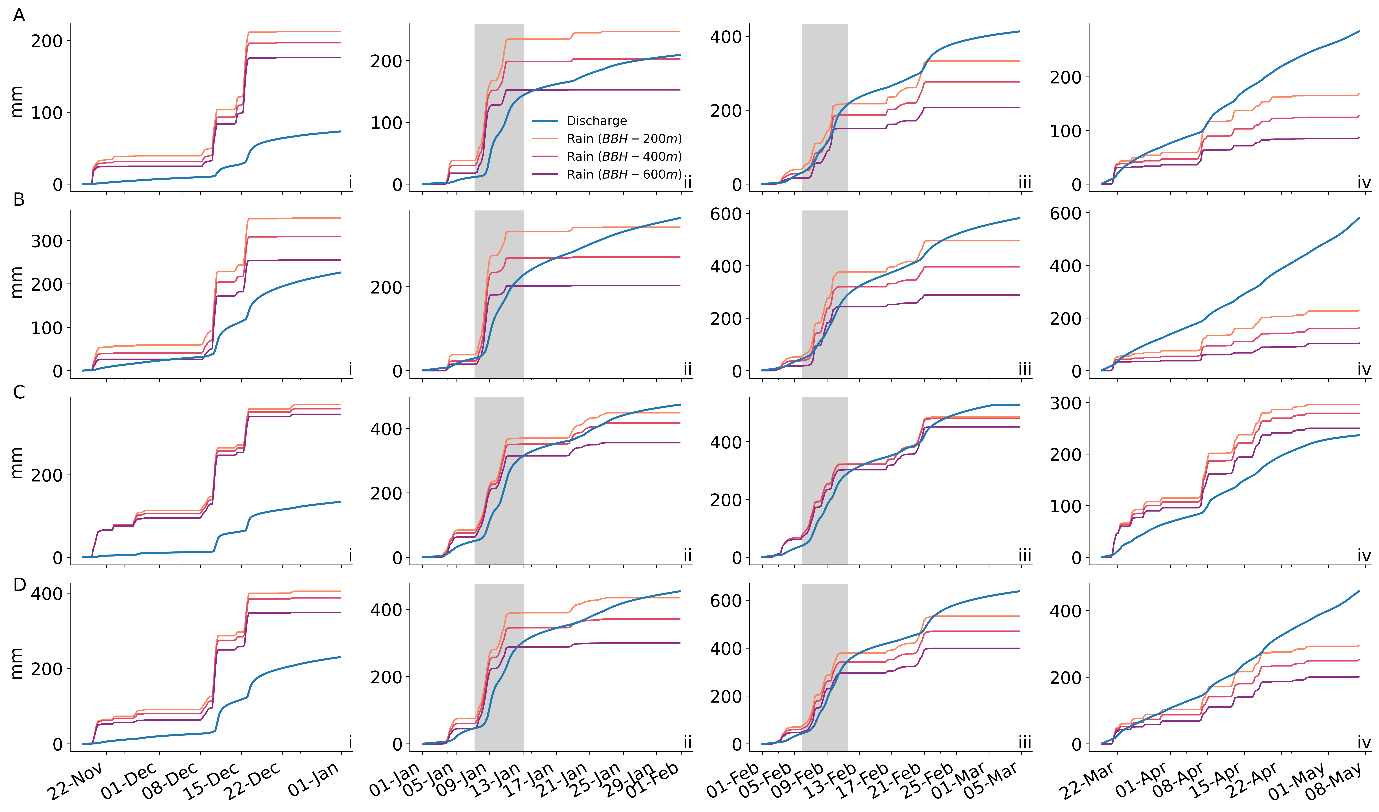


**Fig. S12.** Discharge and estimated rainfall accumulations at subdaily USGS gauges in the (A) central Feather, (B) northern Yuba, (C) southern Yuba, and (D) American. Accumulations are separated by (i) early-season, (ii) January – isolating the 7J event, (iii) February – isolating the 6F event, and (iv) spring periods. Rainfall was partitioned using upwind snow levels, which are systematically lowered here to explore how snow level “bending” affects the rainfall-discharge relationship.


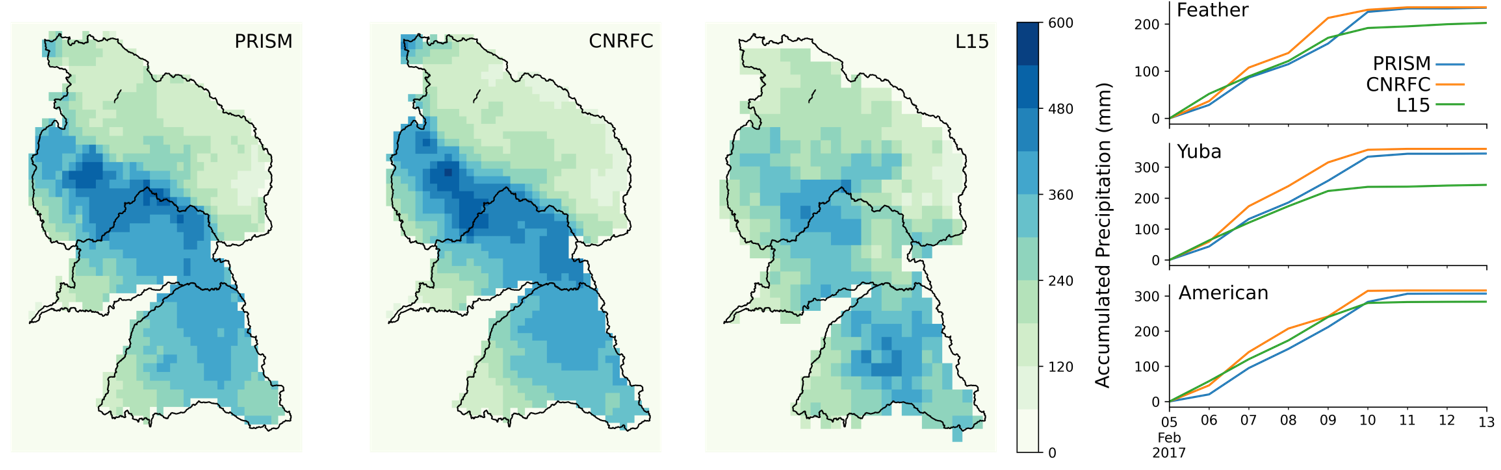


**Fig. S13.** Event total precipitation (6-12 February) over study basins, comparing the Parameter-elevation Regressions on Independent Slopes Model (PRISM^10^, available at https://prism.oregonstate.edu), CNRFC, and Livneh et al.^11^ (L15) products. Watershed average accumulations are shown to the right.

**Table S1.** Comparisons of synoptic and surface conditions over the study basins during the 7 January and 6 February storms when snow levels resided above 1,600 m.

| **Synoptic Metrics** | **7 Jan AR** | **6 Feb AR** | **Δ% (Feb – Jan)** |
| --- | --- | --- | --- |
| High-BBH hours | 58 | 62 | + 6.90% |
| Total Integrated Vapor Transport (10^6^ kg m^-1^) | 82.59 | 85.03 | + 2.95% |
| Total Integrated Heat Transport (10^12^ J m^-1^) | 14,721 | 14,126 | – 4.04% |
| Mean Moist Static Energy Gradient (kJ kg^-1^) | 51.74 | 50.02 | – 3.31% |
| **Station Metrics** | **7 Jan AR** | **6 Feb AR** | **Δ (Feb – Jan)** |
| Median Air Temperature (˚C)  Z < 1,200 m  1,200 m < Z < 1,600 m  1,600 m < Z < 2,000 m  Z > 2,000 m | 6.23 (n=8)  3.89 (n=8)  1.67 (n=10)  – 0.35 (n=10) | 8.89 (n=8)  5.56 (n=12)  3.46 (n=11)  1.13 (n=9) | + 2.66  + 1.67  + 1.79  + 1.48 |
| Median Wind Speed (m s^-1^)  Z < 1,200 m  1,200 m < Z < 1,600 m  1,600 m < Z < 2,000 m  Z > 2,000 m | 3.13 (n=6)  4.47 (n=6)  3.58 (n=4)  4.92 (n=2) | 2.24 (n=6)  3.13 (n=9)  2.23 (n=7)  4.02 (n=1) | – 0.89  – 1.34  – 1.34  – 0.90 |

**Table S2.** Snow pillows used in this study obtained from the California Department of Water Resources’ California Data Exchange Center. Asterisks indicate available snow depth measurements obtained from either CDEC (*) or ARHO (**).

| **Station Name** | **ID** | **River Basin** | **Longitude** | **Latitude** | **Elevation (m)** | **Operator** |
| --- | --- | --- | --- | --- | --- | --- |
| Four Trees | FOR | Feather | -121.32 | 39.81 | 1,586 | CA DWR |
| Bucks Lake | BKL | Feather | -121.25 | 39.85 | 1,790 | CA DWR |
| Harkness Flat | HRK* | Feather | -121.28 | 40.42 | 1,890 | CA DWR |
| Rattlesnake | RTL | Feather | -121.04 | 40.13 | 1,893 | CA DWR |
| Humbug | HMB | Feather | -121.37 | 40.12 | 1,981 | CA DWR |
| Gold Lake | GOL | Feather | -120.62 | 39.67 | 2,057 | CA DWR |
| Pilot Peak | PLP* | Feather | -120.88 | 39.79 | 2,073 | CA DWR |
| Grizzle Ridge | GRZ | Feather | -120.65 | 39.92 | 2,103 | CA DWR |
| Kettle Rock | KTL | Feather | -120.72 | 40.14 | 2,225 | CA DWR |
| Robbs Powerhouse | RBP | American | -120.38 | 38.90 | 1,570 | SMUD |
| Blue Canyon | BLC* | American | -120.71 | 39.28 | 1,609 | USBR |
| Greek Store | GKS* | American | -120.56 | 39.07 | 1,707 | USBR |
| Robbs Saddle | RBB** | American | -120.38 | 38.91 | 1,798 | SMUD |
| Robinson Cow Camp | RCC* | Yuba | -120.68 | 39.62 | 1,975 | CA DWR |
| Huysink | HYS* | American | -120.53 | 39.28 | 2,012 | USBR |
| Van Vleck | VVL** | American | -120.31 | 38.94 | 2,042 | SMUD |
| Central Sierra Snow Lab | CSL | Yuba | -120.37 | 39.33 | 2,103 | NRCS |
| Silver Lake | SIL* | American | -120.12 | 38.68 | 2,164 | USBR |
| Forni Ridge | FRN | American | -120.22 | 38.80 | 2,282 | USBR |
| Alpha | ALP** | American | -120.22 | 38.80 | 2,316 | SMUD |
| Caples Lake | CAP | American | -120.04 | 38.71 | 2,438 | USBR |
| Schneiders | SCN** | American | -120.07 | 38.75 | 2,667 | SMUD |

**Table S3.** Soil moisture measurements were obtained from a variety of sources, which place sensors at different depths beneath the ground surface.

| **Station Name** | **ID** | **River Basin** | **Longitude** | **Latitude** | **Elevation (m)** | **Source** | **Sensor Depths (cm)** |
| --- | --- | --- | --- | --- | --- | --- | --- |
| Alta | ata | American | -120.82 | 39.20 | 1,048 | NOAA | 10, 15 |
| Blue Canyon | blu | American | -120.71 | 39.28 | 1,604 | NOAA | 10 |
| Big Bend | bbd | Yuba | -120.52 | 39.31 | 1,754 | NOAA | 10 |
| Chickering American River Reserve | UCCA | American | -120.36 | 39.25 | 1,841 | WRCC | 5 |
| Central Sierra Snow Lab | CSL | Yuba | -120.37 | 39.33 | 2,103 | NRCS | 5, 20, 50 |
| Alpha | ALP | American | -120.22 | 38.80 | 2,316 | ARHO | 30, 60 |
| Schneiders | SCN | American | -120.07 | 38.75 | 2,675 | ARHO | 30, 60 |

**Table S4.** Surface meteorological stations, organized by river basin and elevation. Note that while some stations are identical to those in Tables S2 and/or S3, the data sources and native timestep (T, minutes) may differ. Binary flags indicate whether PPT (precipitation), TAIR (air temperature), QAIR (humidity), and WV (wind speed) were used in this study. Flags marked with “*” and “**” indicate a variable was screened from analysis during the 7 January and 6 February storms, respectively.

| **Station Name** | **ID** | **River Basin** | **Longitude** | **Latitude** | **Elevation (m)** | **Source** | **PPT** | **TAIR** | **QAIR** | **WV** | **T** |
| --- | --- | --- | --- | --- | --- | --- | --- | --- | --- | --- | --- |
| Mineral | MIN | Sacramento | -121.61 | 40.35 | 1511 | CDEC | 1 | 0 | 0 | 0 | 30 |
| Rice Canyon | RNYC1 | Feather | -120.33 | 39.53 | 2,116 | RAWS | 0 | 1** | 1** | 1** | 60 |
| Grizzle Ridge | GRZ | Feather | -120.65 | 39.92 | 2,103 | CDEC | 1 | 0 | 0 | 0 | 60 |
| Jordan Peak | JDP | Feather | -120.29 | 40.04 | 2,076 | CDEC | 0 | 1 | 1 | 1* | 60 |
| Harkness Flat | HRK | Feather | -121.28 | 40.42 | 1,890 | CDEC | 0 | 1 | 1 | 0 | 60 |
| Swain Mountain | SWNC1 | Feather | -121.10 | 40.45 | 1,859 | RAWS | 1 | 1 | 1 | 1 | 60 |
| Lake David | DAV | Feather | -120.47 | 39.88 | 1,758 | CDEC | 0 | 0 | 0 | 0 | 60 |
| Doyle Crossing | DOY | Feather | -120.48 | 40.12 | 1,728 | CDEC | 0 | 1 | 0 | 1 | 60 |
| Coyote | CYVC1 | Feather | -120.48 | 39.99 | 1,698 | RAWS | 0 | 1 | 1 | 1 | 60 |
| Thompson Valley | TVL | Feather | -120.48 | 39.98 | 1,647 | CDEC | 0 | 1 | 1 | 1* | 60 |
| Westwood | WWD | Feather | -120.90 | 40.31 | 1,570 | CDEC | 0 | 1 | 1 | 1 | 60 |
| Mohawk / Denten Creek | MWKC1 | Feather | -120.59 | 39.78 | 1,561 | RAWS | 0 | 1* | 1* | 1* | 60 |
| Plumas Eureka St Park Weather Station | EWS | Feather | -120.70 | 39.76 | 1,557 | CDEC | 1 | 0 | 0 | 0 | 60 |
| La Porte | LAP | Feather | -120.98 | 39.68 | 1,518 | CDEC | 1 | 0 | 0 | 0 | 60 |
| Sierraville | SVL | Feather | -120.37 | 39.58 | 1,516 | CDEC | 0 | 0 | 0 | 0 | 60 |
| Hamilton Branch | HAM | Feather | -121.09 | 40.27 | 1,390 | CDEC | 1 | 0 | 0 | 0 | 15 |
| Cashman | CSH | Feather | -120.92 | 40.00 | 1,378 | CDEC | 1 | 1 | 1 | 1 | 60 |
| Pratville (PG&E) | PVL | Feather | -121.16 | 40.21 | 1,378 | CDEC | 0 | 1* | 1* | 0 | 60 |
| Strawberry Valley | SBY | Feather | -121.11 | 39.56 | 1,161 | CDEC | 1 | 0 | 0 | 0 | 30 |
| Greenville | GRE | Feather | -120.94 | 40.14 | 1,085 | CDEC | 1 | 0 | 0 | 0 | 15 |
| Brush Creek | BRS | Feather | -121.34 | 39.69 | 1,085 | CDEC | 1 | 0 | 0 | 0 | 60 |
| Tay Nelson St Taylorsville | TAY | Feather | -120.84 | 40.08 | 1,079 | CDEC | 0 | 1 | 1 | 1 | 60 |
| Stirling City | SRL | Feather | -121.53 | 39.90 | 1,073 | CDEC | 1 | 0 | 0 | 0 | 30 |
| Quincy Ranger District | QYR | Feather | -120.94 | 39.98 | 1,067 | CDEC | 1 | 1 | 1 | 1 | 60 |
| Quincy | QCY | Feather | -120.95 | 39.94 | 1,039 | CDEC | 1 | 0 | 0 | 0 | 60 |
| Jarbo Gap | JAR | Feather | -121.49 | 39.74 | 823 | CDEC | 1 | 1 | 1 | 1 | 60 |
| Central Sierra Snow Lab | CSL | Yuba | -120.37 | 39.33 | 2,103 | WRCC | 0 | 1 | 1 | 0 | 10 |
| Big Bend | bbd | Yuba | -120.52 | 39.31 | 1,754 | NOAA | 0 | 1 | 1 | 1* | 2 |
| Bowman Lake | BOL | Yuba | -120.65 | 39.45 | 1,643 | CDEC | 1 | 0 | 0 | 0 | 15 |
| Lake Spaulding (PG&E) | LSP | Yuba | -120.63 | 39.32 | 1,571 | CDEC | 1 | 1* | 1* | 0 | 15 |
| Sierra City | SRC | Yuba | -120.65 | 39.57 | 1,432 | CDEC | 1 | 0 | 0 | 0 | 15 |
| Deer Creek Forebay | DRC | Yuba | -120.83 | 39.30 | 1,358 | CDEC | 0 | 1 | 1 | 0 | 15 |
| White Cloud | WTC | Yuba | -120.84 | 39.32 | 1,317 | CDEC | 1 | 1 | 1 | 1 | 60 |
| Pike County | PKC | Yuba | -121.20 | 39.48 | 1,132 | CDEC | 1 | 1 | 1 | 1 | 60 |
| Downieville | DNV | Yuba | -120.83 | 39.56 | 890 | CDEC | 1 | 0 | 0 | 0 | 15 |
| Grass Valley | GVY | Yuba | -121.07 | 39.21 | 731 | CDEC | 1 | 0 | 0 | 0 | 15 |
| Bullards Bar | BUD | Yuba | -121.14 | 39.40 | 640 | CDEC | 1 | 0 | 0 | 0 | 15 |
| Schneiders | SCN | American | -120.07 | 38.75 | 2,675 | ARHO | 0 | 1 | 1 | 0 | 15 |
| Mt Lincoln | MTL | American | -120.33 | 39.29 | 2,544 | ARHO | 0 | 1 | 1 | 0 | 15 |
| Caples Lake | CAP | American | -120.04 | 38.71 | 2,439 | ARHO | 0 | 1 | 1 | 1** | 60 |
| Alpha | ALP | American | -120.22 | 38.80 | 2,316 | ARHO | 0 | 1 | 1 | 0 | 15 |
| Duncan Peak | DPK | American | -120.51 | 39.15 | 2,126 | ARHO | 0 | 1 | 1 | 0 | 15 |
| Van Vleck | VVL | American | -120.31 | 38.94 | 2,042 | ARHO | 0 | 1 | 1 | 0 | 15 |
| Dolly Rice | DOR | American | -120.37 | 39.15 | 2,006 | ARHO | 0 | 1 | 1 | 0 | 15 |
| Chickering American River Reserve | UCCA | American | -120.36 | 39.25 | 1,841 | WRCC | 0 | 1 | 1 | 1* | 10 |
| Robbs Saddle | RBB | American | -120.38 | 38.91 | 1,816 | ARHO | 0 | 1 | 1 | 0 | 15 |
| Talbot Camp | TLC | American | -120.38 | 39.19 | 1,741 | ARHO | 0 | 1 | 1 | 0 | 15 |
| Sugarloaf | SKBC1 | American | -120.31 | 38.78 | 1,723 | RAWS | 0 | 1* | 1* | 0 | 60 |
| Blue Canyon | blu | American | -120.71 | 39.28 | 1,604 | NOAA | 1 | 1 | 1 | 1 | 2 |
| Bear Trap | BTP | American | -120.58 | 39.09 | 1,590 | CDEC | 0 | 1 | 1 | 1* | 60 |
| Bald Mountain | BMT | American | -120.68 | 38.90 | 1,426 | CDEC | 1 | 1 | 1 | 1 | 60 |
| Hell Hole | HLH | American | -120.42 | 39.07 | 1,396 | CDEC | 1 | 1 | 1 | 1 | 60 |
| Owens Camp | OWC | American | -120.25 | 38.73 | 1,372 | CDEC | 0 | 1* | 1* | 1* | 60 |
| Seed Orchard RAWS Near Michigan Bluff | SOM | American | -120.73 | 39.09 | 1,311 | CDEC | 1 | 1 | 1 | 1 | 60 |
| Fresh Pond | FPD | American | -120.54 | 38.76 | 1,149 | CDEC | 1 | 0 | 0 | 0 | 15 |
| Alta | ata | American | -120.82 | 39.20 | 1,048 | NOAA | 1 | 1 | 1 | 0 | 2 |
| Placerville Airport | KPVF | American | -120.75 | 38.72 | 788 | NWS | 1 | 1 | 1 | 1 | 20 |
| Drum Power House | DPH | Bear | -120.77 | 39.26 | 1,036 | CDEC | 0 | 1 | 1 | 0 | 15 |
| Secret Town | SRT | Bear | -120.88 | 39.18 | 829 | CDEC | 1 | 1 | 1 | 1 | 60 |
| Bear River at Rollins Reservoir | BRE | Bear | -120.95 | 39.13 | 593 | CDEC | 1 | 0 | 0 | 0 | 15 |

**Table S5.** USGS streamflow gauges used in this study. “*” indicates available 15-minute data.

| **Station Name** | **USGS ID** | **River Basin** | **Longitude** | **Latitude** | **Drainage Area (km^2^)** |
| --- | --- | --- | --- | --- | --- |
| Spanish Creek above Blackhawk Creek at Keddie | 11402000* | Feather | -120.95 | 40.00 | 475 |
| Butt Creek below Almanor-Butt Creek Tunnel near Pratville | 11400500 | Feather | -121.19 | 40.19 | 168 |
| Deer Creek near Smartville | 11418500* | Yuba | -121.27 | 39.22 | 219 |
| North Yuba River below Goodyears Bar | 11413000* | Yuba | -120.94 | 39.52 | 648 |
| South Yuba River at Jones Bar near Grass Valley | 11417500 | Yuba | -121.10 | 39.29 | 820 |
| North Fork American River at North Fork Dam | 11427000* | American | -121.02 | 38.94 | 883 |
| Middle Fork American River above Middle Fork Powerhouse near Foresthill | 11427760 | American | -120.60 | 39.03 | 227 |
| South Fork American River near Kyburz (river only) | 11439500 | American | -120.33 | 38.76 | 500 |
| Rock Creek near Placerville | 11444201 | American | -120.78 | 38.79 | 189 |

**Table S6.** NOAA FMCW snow level radars used in this study. Data are recorded at 10-minute intervals.

| **Station Name** | **ID** | **Longitude** | **Latitude** | **Elevation (m)** |
| --- | --- | --- | --- | --- |
| Oroville | ovl | -121.49 | 39.53 | 114 |
| Colfax | cff | -120.94 | 39.08 | 644 |

**SI References**

1. Bales RC, Cui G, Rice R, Meng X, Zhang Z, Hartsough P, Glaser SD, Conklin MH. Snow depth, air temperature, humidity, soil moisture and temperature, and solar radiation data from the basin-scale wireless-sensor network in American River Hydrologic Observatory (ARHO) (Accessed on 10-Apr-2021). 2020. doi:10.6071/M39Q2V

2. DeWalle DR, Rango A. Principles of Snow Hydrology. 1st ed. New York: Cambridge University Press, New York; 2008. doi:10.1017/CBO9780511535673

3. Malek SA, Glaser SD, Bales RC. Wireless Sensor Networks for Improved Snow Water Equivalent and Runoff Estimates. IEEE Access. 2019;7:18420–18436. doi:10.1109/ACCESS.2019.2895397

4. Hersbach H, Bell B, Berrisford P, Hirahara S, Horányi A, Nicolas J, Peubey C, Radu R, Bonavita M, Dee D, et al. The ERA5 global reanalysis. Quarterly Journal of the Royal Meteorological Society. 2020;146(730):1999–2049. doi:10.1002/qj.3803

5. Henn B, Musselman KN, Lestak L, Ralph FM, Molotch NP. Extreme Runoff Generation From Atmospheric River Driven Snowmelt During the 2017 Oroville Dam Spillways Incident. Geophysical Research Letters. 2020;47(14):e2020GL088189. doi:10.1029/2020GL088189

6. Muñoz Sabater J. ERA5-Land hourly data from 1981 to present. Copernicus Climate Change Service (C3S) Climate Data Store (CDS). (Accessed on 30-Sep-2021). 2019. doi:10.24381/cds.e2161bac

7. Masuda M, Yatagai A, Kamiguchi K, Tanaka K. Daily Adjustment for Wind-Induced Precipitation Undercatch of Daily Gridded Precipitation in Japan. Earth and Space Science. 2019;6(8):1469–1479. doi:10.1029/2019EA000659

8. Hatchett BJ. Snow level characteristics and impacts of a spring typhoon-originating atmospheric river in the Sierra Nevada, USA. Atmosphere. 2018;9(6):233. doi:10.3390/atmos9060233

9. Hatchett BJ, Cao Q, Dawson PB, Ellis CJ, Hecht CW, Kawzenuk B, Lancaster JT, Osborne TC, Wilson AM, Anderson ML, et al. Observations of an Extreme Atmospheric River Storm With a Diverse Sensor Network. Earth and Space Science. 2020;7(8). doi:10.1029/2020EA001129

10. Daly C, Neilson RP, Phillips DL. A statistical-topographic model for mapping climatological precipitation over mountainous terrain. Journal of Applied Meteorology and Climatology. 1994;33(2):140–158. doi:10.1175/1520-0450(1994)033<0140:ASTMFM>2.0.CO;2

11. Livneh B, Bohn TJ, Pierce DW, Munoz-Arriola F, Nijssen B, Vose R, Cayan DR, Brekke L. A spatially comprehensive, hydrometeorological data set for Mexico, the U.S., and Southern Canada 1950-2013. Scientific Data. 2015;2:150042. doi:10.1038/sdata.2015.42
